# Targeting cuproptosis with nano material: new way to enhancing the efficacy of immunotherapy in colorectal cancer

**DOI:** 10.3389/fphar.2024.1451067

**Published:** 2024-12-03

**Authors:** Xiangdong Liu, Wanqiu Zhang, Shaozhong Wei, Xinjun Liang, Bo Luo

**Affiliations:** ^1^ Department of Radiotherapy Center, Hubei Cancer Hospital, The Seventh Clinical School Affiliated of Tongji Medical College, Huazhong University of Science and Technology, Wuhan, China; ^2^ Hubei Provincial Clinical Research Center for Colorectal Cancer, Wuhan, China; ^3^ Wuhan Clinical Research Center for Colorectal Cancer, Wuhan, China; ^4^ Department of Gastrointestinal Oncology Surgery, Hubei Cancer Hospital, Tongji Medical College, Huazhong University of Science and Technology, Wuhan, China; ^5^ Department of Abdominal Oncology, Hubei Cancer Hospital, Tongji Medical College, Huazhong University of Science and Technology, Wuhan, China

**Keywords:** colorectal cancer, cuproptosis, immunotherapy, nanoparticle, targeted therapy

## Abstract

Colorectal cancer has emerged as one of the predominant malignant tumors globally. Immunotherapy, as a novel therapeutic methodology, has opened up new possibilities for colorectal cancer patients. However, its actual clinical efficacy requires further enhancement. Copper, as an exceptionally crucial trace element, can influence various signaling pathways, gene expression, and biological metabolic processes in cells, thus playing a critical role in the pathogenesis of colorectal cancer. Recent studies have revealed that cuproptosis, a novel mode of cell death, holds promise to become a potential target to overcome resistance to colorectal cancer immunotherapy. This shows substantial potential in the combination treatment of colorectal cancer. Conveying copper into tumor cells via a nano-drug delivery system to induce cuproptosis of colorectal cancer cells could offer a potential strategy for eliminating drug-resistant colorectal cancer cells and vastly improving the efficacy of immunotherapy while ultimately destroy colorectal tumors. Moreover, combining the cuproptosis induction strategy with other anti-tumor approaches such as photothermal therapy, photodynamic therapy, and chemodynamic therapy could further enhance its therapeutic effect. This review aims to illuminate the practical significance of cuproptosis and cuproptosis-inducing nano-drugs in colorectal cancer immunotherapy, and scrutinize the current challenges and limitations of this methodology, thereby providing innovative thoughts and references for the advancement of cuproptosis-based colorectal cancer immunotherapy strategies.

## 1 Introduction

Colorectal cancer (CRC) is a globally prevalent malignancy, ranking third in incidence and second in terms of cancer-related mortality (Sung et al., 2021). At the time of initial diagnosis, distant metastases are observed in approximately 20–50 percent of colorectal cancer patients (Cervantes et al., 2023), leading to a discouraging 5-year survival rate of merely 12 percent (Xiong et al., 2019). Given the aggressive nature and poor prognosis associated with metastatic colorectal cancer, physicians and researchers face significant challenges in its management and treatment. Conventional treatment methods for CRC encompass endoscopic therapy, surgery, radiotherapy, local therapy, as well as systemic therapy utilizing chemotherapeutic agents. Programed cell death protein 1 (PD-1), also known as PDCD1 and CD279, is expressed on the surface of activated T cells, B cells, NK cells and other immune cells. PD-L1 and PD-L2 are ligands of PD-1, which are widely expressed in antigen-presenting cells, tumor cells and a variety of tissues. When PD-1 present on the surface of T cells interacts with PD-L1 expressed by cancer cells, it can inhibit T cell activation and exert an immunosuppressive function (Lin et al., 2024). On the surface of tumor cells, PD-L1 is overexpressed to evade CD8 + T cell-mediated cytotoxicity, thereby facilitating tumor immune evasion. The anti-PD-1/PD-L1 antibodies serve as potent immune checkpoint inhibitors (ICIs) are designed to block the activity of immune checkpoint proteins and promote T cell activation to achieve anti-tumor immune effects. In 2017, pembrolizumab, a PD-1 inhibitor, was approved by the U.S. Food and Drug Administration for solid tumor treatment, marking a milestone in immunotherapy for cancer. However, the therapeutic efficacy of immunotherapy in colorectal cancer patients is limited to approximately 15% of cases characterized by mismatch repair deficiency (dMMR) and high microsatellite instability (MSI-H), referred to as dMMR/MSI-H subtypes (Ding et al., 2023).Besides, immunotherapy offers minimal clinical benefits for the vast majority (over 95 percent) of metastatic colorectal cancer patients outside these subgroups (Franke et al., 2019). Therefore, it is imperative to explore new targets and techniques to overcome resistance and completely eradicate colorectal tumors through immunotherapy.

Copper, serving as an essential cofactor for enzyme activity, plays a vital role in various metabolic processes such as energy metabolism, mitochondrial respiration, antioxidant defense, and biosynthesis (Ge et al., 2022). The serum copper concentration in healthy adults ranges from 0.7 to 1.4 ug/mL (Lutsenko et al., 2024). To maintain cellular health and avoid toxicity, the concentration of free copper in the cytoplasm varies from 10^−15^ M to 10^−21^ M (Wu et al., 2023). Typically, the intracellular concentration of free copper ions is approximately 10^−18^ M, representing less than one free copper ion per cell (Rae et al., 1999). Elevated levels of copper have been observed in tumor tissues and serum of patients with breast, lung, and colorectal cancer (Shanbhag et al., 2021). Research indicates that the copper concentration within normal colorectal tissue is 8.3 ± 0.3 μM, whereas it dramatically escalates in colorectal cancer tissue to 22 ± 3 μM (Kang et al., 2014). These elevated serum copper levels exhibit a strong correlation with tumor stage and disease progression. For example, Baszuk et al. reported that 62% of patients with stage I-II colorectal cancer exhibited blood copper levels within the highest quartile, and blood copper levels exceeding 0.93 μg/mL were correlated with a heightened prevalence of colorectal cancer in the population (Baszuk et al., 2021).Recently, Tsvetkov et al. found that pulse treatment with the copper ionophore elesclomol-Cu (1:1 ratio) at concentrations as low as 40 nM for only 2 h resulted in a 15- to 60-fold increase in intracellular copper levels that triggered cell death. Then, they identified a novel form of non-regulatory cell death known as cuproptosis that is dependent on mitochondrial metabolism (Tsvetkov et al., 2022). Inducing cuproptosis in tumor cells presents an innovative approach for the treatment of colorectal cancer and has the potential to enhance the efficacy of CRC immunotherapy. Firstly, key cuproptosis genes are associated with immune-related genes expression and infiltration of immune cells within the tumor microenvironment (TME) (Guo P. et al., 2023). Secondly, it has been found that elevated intracellular copper can upregulate PD-L1 expression by inducing EGFR phosphorylation within tumors *in vivo* (Voli et al., 2020). Thus, by employing copper ionophores (a kind of compounds capable of selectively binding to copper ions and transporting them into cells or specific organelles, such as DSF, ES), it becomes feasible to upregulate PD-L1 expression in tumor cells, thereby transforming colorectal cancer from a “cold tumor” into an immunologically responsive “hot tumor” and enhancing the efficacy of immunotherapy. The US FDA has endorsed PD-L1 expression in tumor cells as a predictive biomarker for responsiveness to anti-PD-1/PD-L1 therapies (Li H. et al., 2022). While increased PD-L1 expression within tumors promotes tumor evasion, tumoral PD-L1 expression reflects an immunologically active environment that amplifying the impact of ICIs. “Hot” tumors exhibit elevated levels of immune cell infiltration, particularly cytotoxic T cells and a pro-inflammatory microenvironment, whereas “cold” tumors exhibit decreased levels of immune cell infiltration and an immunosuppressive microenvironment. Generally, “hot” tumors respond more favorably to ICIs due to their pre-existing immune activity and augmented expression of immune checkpoint molecules, such as PD-1 and PD-L1 (Ortega et al., 2024).For instance, Zhou et al. found that DSF/Cu can upregulate PD-L1 expression in hepatocellular carcinoma cells by inhibiting PARP1 and enhancing GSK3β phosphorylation at Ser9 point, and combination therapy with DSF/Cu and an anti-PD-1 antibody showed much better antitumor efficacy than monotherapy (Zhou et al., 2019). Consequently, the combination of cuproptosis-inducing strategies and immunotherapy exhibits promising prospects.

While the induction of copper-mediated cell death in colorectal cancer cells relies heavily on the targeted transportation of copper via copper ionophores, current small molecule-based copper ionophores suffer from limitations such as short blood half-life, limited transport capacity, and inadequate tumor targeting (Oliveri, 2022). To efficiently and accurately deliver more copper to tumor cells, nanomaterial-based drug delivery systems can be employed. The integration of nanomaterials can enhance the accumulation of copper ionophores at the target tumor tissues while minimizing harm to healthy tissues, thereby augmenting the therapeutic efficacy of copper ionophores. Furthermore, combining cuproptosis-inducing strategies with other anti-tumor approaches like immunotherapy, photothermal therapy, photodynamic therapy, and chemodynamic therapy could further potentiate their therapeutic effectiveness. Recently, the combined immunotherapy of cuproptosis-inducing nanoparticles has garnered significant attention and demonstrated remarkable synergistic anti-tumor efficacy, emerging as a rapidly evolving frontier in cancer treatment. The advent of nanotechnology presents an immense opportunity for advancing the combined immunotherapy of cuproptosis-induced drugs, offering substantial advantages in the clinical management of colorectal cancer.

## 2 The association between copper and colorectal cancer

### 2.1 Systemic and cellular copper homeostasis

Copper is an essential micronutrient for human survival, playing crucial roles in diverse protein synthesis and functioning as a vital modulator of intracellular signaling and gene expression. However, excessive copper levels can lead to irreparable cellular damage and potentially result in disorders such as Wilson disease (Bandmann et al., 2015). Therefore, the concentration of copper within human physiology typically remains within a narrow range (0.7–1.4 ug/mL) in serum (Lutsenko et al., 2024), constantly balanced dynamically between the cooperative efforts of the gut and liver. Humans obtain copper primarily from food, and the median daily dietary copper intake for adults has been reported to be 1.0–1.6 mg (Myint et al., 2018). The duodenum and small intestine serve as the primary sites for copper assimilation. Upon entry into the intestine, Cu^2+^ is converted to Cu^+^ by metalloreductases like prostatic six-transmembrane epithelial antigen (STEAP) and duodenal cytochrome b (DCYTB) in the apical membrane of intestinal epithelial cells due to the fact that CTR1 can only transport Cu ^+^. Moreover, Cu^2+^ can also be directly imported into cells through DMT1, however, these copper ions can’t be directly utilized by cells, and research has indicated that DMT1 is not essential for intestinal copper transport (Shawki et al., 2015). Cu^+^ is then transported into cells through the copper transporter CTR1 located at the apex of intestinal epithelial cells before being secreted into the blood via ATPase ATP7A activity (Guan et al., 2023). In intestinal cells, a substantial number of ATP7A migrate to the basolateral membrane and accept Cu^+^ from ATOX1, facilitating the transfer of Cu^+^ from intestinal cells to blood (Kaler, 2011). Once Cu^+^ leave intestinal cells, the oxidative environment of the interstitial fluid can convert Cu^+^ into Cu^2+^. Copper entering the bloodstream can bind to various significant proteins, such as ceruloplasmin (CP) and albumin. In human serum, the majority of copper exists in the Cu^2+^ state and binds primarily to ceruloplasmin (Linder and Hazegh-Azam, 1996). Ceruloplasmin is a multifunctional iron oxidase that plays a pivotal role in copper transport and storage. It is the most significant copper-carrying protein in plasma. Copper bound to CP accounts for the vast majority of serum copper content, approximately 90%, and the remaining copper ions bind to albumin or free amino acids in serum (Grochowski et al., 2019). Then, Cu^+^ is transported through the portal vein to the liver, metabolized or stored in hepatic cells by interacting with metallothioneins MT1 and MT2 (Bian et al., 2023). The liver can also utilize copper to synthesize and secrete Cu-containing ceruloplasmin and release it into the blood. Following this, copper ions can be retrieved by other tissues through the blood circulation (Munk et al., 2023). Most of the copper released from the liver re-enters circulation through ATP7B and once again binds to plasma proteins such as ceruloplasmin and albumin for transportation to specific organs or tissues where it exerts its function (Chen et al., 2020). Most copper must be returned to the liver for elimination via the bile, which serves as the predominant mechanism for copper excretion from the body. Bile copper possesses diminished reabsorption potential, and its absorbability varies with the amount of copper in liver cells. Generally speaking, copper homeostasis within the human body is predominantly controlled by the level and form of copper excretion via the bile (Linder, 2001). Nonetheless, copper is expelled not solely through the bile. Indeed, up to 4.5 mg of copper is secreted into the gastrointestinal tract on a daily basis in adults, with the majority being reabsorbed (Linder et al., 1998). Large fragments of ceruloplasmin (high copper content and resistant to proteolysis) may provide a means of excreting copper that is not reabsorbed by the intestine (Chowrimootoo and Seymour, 1994).Furthermore, the kidney is also one of the pathways for eliminating excess copper (Linder et al., 1998).Part of the filtered copper is reabsorbed by renal tubules, and the rest is excreted with urine.

The cytoplasmic copper level is tightly regulated by a variety of proteins, including copper enzymes, copper chaperones, and membrane transporters (Wen et al., 2021). Copper possesses two primary oxidation states. Within cells, copper primarily exists as Cu^+^, while outside the cell it is predominantly Cu^2+^. They participate the intracellular reduction and extracellular oxidation reactions, respectively. The standard redox potential of Cu^2+^/Cu^+^ is 0.153 V (Palumaa, 2013). Under physiological conditions, Cu^2+^ and Cu^+^ are capable of accepting and donating electrons, switching between the two oxidation states (Cu^2+^ and Cu^+^), thereby facilitating electron transfer between molecules and contributing significantly to O_2_ transport, respiratory regulation, nerve cell differentiation, and signaling (Kardos et al., 2018). The capacity of copper to cyclically transition through redox reactions between Cu^2+^ and Cu^+^ results in redox potentials for copper-enriched enzymes typically between +0.25 and +0.75 V, thereby permitting the generation of electrons from diverse substrates, such as catechol, superoxide, ascorbic acid, and iron (Ladomersky and Petris, 2015). The balance between Cu^2+^ and Cu^+^ is crucial for preserving cellular redox potentials, and it's tightly regulated by various intracellular proteins, including superoxide dismutase (SOD), catalase, and glutathione peroxidase. Besides, copper-binding proteins can also influence the balance between Cu^2+^ and Cu^+^. These proteins can bind copper ions and regulate their oxidation status. For example, metallothionein could bind both Cu^2+^ and Cu^+^ and play a significant role in maintaining copper homeostasis (Krężel and Maret, 2017).During the reduction of Cu^2+^ to Cu^+^, reactive oxygen species (ROS), including superoxide anion (O_2_^−^), nitric oxide (NO^−)^, hydroxyl radical (OH^−^), and hydrogen peroxide (H_2_O_2_), can be produced via the Fenton reaction (Liochev and Fridovich, 2002). These ROS can subsequently interact with transition metals such as Cu^+^ to produce highly reactive hydroxyl radicals. Hydroxyl radicals can cause significant cellular oxidative damage due to their high standard reduction potential of 2.8 V (Wang and Zhang, 2018). Moreover, ROS can also influence the activity of antioxidant enzymes and copper-binding proteins. ROS can not only deactivate antioxidant enzymes and diminish their capacity to maintain redox potential, but also modify copper-binding proteins and alter their affinity for copper ions, thereby impacting the balance between Cu^2+^/Cu^+^ and redox potential, leading to an increase in the oxidation state of copper and a shift in redox potential (Vo et al., 2024).

CTR1 predominantly facilitates the entry of Cu^+^ into intestinal epithelial cells, which plays a crucial role in promoting the absorption of copper in the intestine (Lutsenko, 2021). CTR1 is a homotrimeric membrane protein. Each monomer incorporates an extracellular N-terminal domain and is essential for copper interaction (Gupta and Lutsenko, 2009). Alterations in intracellular copper concentration can trigger CTR1 to relocate between the plasma membrane and intracellular vesicles. Augmented copper levels can endocytose CTR1 from the plasma membrane, thereby preventing copper overload. After cellular uptake, Cu^+^ can maintain extremely low intracellular free copper concentrations by binding to MT1/2 and GSH, protecting Cu^+^ from disproportionation and then transferring Cu^+^ to different copper chaperones (Tang et al., 2023). Free copper is extraordinarily reactive. Be it in serum or within cells, copper binds to various molecules, thus no free copper exists. A large number of Cu-binding proteins are reside in the cytoplasm, forming an exchangeable “Cu pool” (Kim et al., 2023). Cu^+^ could interacts with various copper chaperones such as COX17, ATOX1, CCS, and SOD1 before being directed to specific subcellular compartments like mitochondria, trans-Golgi network (TGN), and nucleus (Chen et al., 2022). Cytosolic Cu^+^ can be transported to the mitochondrial membrane space (IMS) via copper ligands (CuL) and subsequently into the mitochondrial matrix via solute carrier family 25 member 3 (SLC25A3) of the inner mitochondrial membrane (IMM) (Tian et al., 2023). Cox17 shuttles between the cytoplasm and the mitochondrial membrane gap. It not only conveys Cu^+^ from the cytoplasm to SCO1 or SCO2 located on the inner mitochondrial membrane, but also transports Cu^+^ from the cytoplasm to Cox11, and subsequently emits Cu^+^ to the CuA and CuB sites of Cox1 and COX2, respectively (Chojnacka et al., 2015). COX1 and COX2, as two subunits of CcO, are indispensable copper-dependent enzymes in the oxidative phosphorylation process. When combined with Cu^+^, they can activate the oxidative respiratory chain within mitochondria (Nývltová et al., 2022). ATOX1 transfers Cu^+^ to the metal-binding sites of ATP7A and ATP7B in the TGN while promoting the synthesis of copper-dependent proenzymes such as lysine oxidase and tyrosinase (Lutsenko et al., 2007). Additionally, ATOX1 shuttles Cu^+^ to the nucleus where it modulates gene expression through transcription factor binding (Itoh et al., 2008). CCS facilitates the delivery of copper ions to SOD1 which utilizes them for catalyzing superoxide disproportionation in the cytoplasm, thus mitigating ROS-induced damage outside mitochondria and maintaining intracellular Cu ion homeostasis (Boyd et al., 2020). Copper transporters ATP7A and ATP7B are responsible for the cellular excretion of excess copper (Ruturaj et al., 2024). ATP7A and ATP7B are located on the TGN or plasma membrane and pump Cu^+^ from ATOX1 to the other side of the membrane. Elevated intracellular Cu levels can augment ATOX1-mediated Cu ion delivery to ATP7A and ATP7B and promote ATP7A and ATP7B movement from the TGN to the plasma membrane (Cox and Moore, 2002). Specifically, when intracellular copper levels are increased, on the one hand, ATP7A will move from the TGN to the plasma membrane and facilitate intracellular copper excretion (Linder, 2001). Studies have shown that ATP7A expression increases in multiple tissues such as intestine, heart, and spleen to efficiently export more copper after excessive copper intake in humans (Sailer et al., 2024). On the other hand, elevated cellular copper concentration also causes ATP7B to redistribute to vesicles and apical vacuoles in the apical membrane of hepatocytes adjacent to the bile ducts and promotes biliary excretion of excess copper in the liver (Cater et al., 2006). ATP7B, which encodes the WND p-type ATPase and is predominantly expressed in the liver (Hasan et al., 2012), is essential for biliary excretion of Cu and binding of Cu to hepatic ceruloplasmin, and is predominantly localized to TGN at low extracellular copper concentrations (<1 μmol/L) (Tapiero et al., 2003). The copper transit to the bile involves HAH1/ATOX1, WND, and exocytosis or trafficking of WND to the brush border of the bile canaliculus (Roelofsen et al., 2000). Moreover, copper excretion may require additional intracellular liver copper-binding proteins COMMD1 (the MURR1 domain of copper metabolism) and XIAP (X-linked inhibitor of apoptosis proteins) (Prohaska, 2008). These intricate mechanisms underline the critical importance of maintaining appropriate cellular copper homeostasis for optimal cell functionality.

### 2.2 Copper and colorectal cancer signaling pathway

Copper exhibits the ability to bind and activate important molecules involved in multiple transduction pathways within colorectal cancer cells, demonstrating a strong association with various malignant phenotypes observed in colorectal tumors. This includes the activation of a cascade of receptor kinases. For example, copper can facilitate the binding and stimulation of tyrosine phosphorylation in RTKs, which play a key role in signal transduction pathways associated with RTK (He et al., 2019). Subsequent activation of RTKs leads to downstream phosphorylation events involving extracellular regulated protein kinase ERK and tyrosine kinase AKT, promoting migratory and proliferative activities in colorectal cancer cells (Vitaliti et al., 2022). Additionally, copper activates various core kinases that contribute significantly to the initiation and progression of colorectal tumors. Within the mitogen-activated protein kinase (MAPK) pathway, MEK1/2 serves as an essential regulator for processes such as tumor cell proliferation, apoptosis, differentiation, and metabolism. Copper ions can stimulate the MAPK pathway through MEK1/2 (Gao and Zhang, 2023) while simultaneously engaging autophagic signaling via interaction with ULK1/2 proteins (Hu et al., 2021), suggesting potential pharmacological strategies for targeting these signaling networks and overcoming colorectal cancer drug resistance. The BRAF^V600E^ alteration serves as a robust prognostic indicator for metastatic colorectal cancer, while copper ions can further enhance colon carcinogenesis by triggering the BRAF^V600E^-MEK-MAPK signaling axis (Brady et al., 2017). Additionally, the WNT signaling pathway plays a crucial role in maintaining the stemness characteristics of colorectal cancer stem cells. Recent research suggests that copper interacts with PDK1 and facilitates its binding to AKT, consequently triggering the WNT/β-catenin pathway and enhancing CSC properties. Further investigations unveiled that the β-catenin/TCF4 transcriptional complex directly interacts with the ATP7B promoter, thereby inducing its expression, reducing intracellular copper and inhibiting cuproptosis (Liu YT. et al., 2024). Furthermore, it has been revealed that interleukin-17 (IL-17)-driven STEAP4-dependent cellular copper accumulation is critical for sustaining nuclear factor kappa B (NF-κB) pathway activation and enhancing XIAP activity, which ultimately promoting colorectal cancer development and multidrug resistance (Liao et al., 2020). Therefore, copper exerts significant influence on colorectal cancer-associated signaling pathway, providing potential insights into our understanding and therapeutic options for this disease.

### 2.3 Copper and colorectal cancer progression and metastasis

Copper, an essential cofactor of cytochrome c oxidase within mitochondria, plays a vital role in meeting the energy demands of rapidly proliferating cells. Consequently, cancer cells require higher levels of copper compared to non-dividing cells (Lopez et al., 2019). Copper can stimulate cellular growth and proliferation through various signaling pathways, known as copper hyperplasia, and also can promote cancer progression by activating metastasis-associated enzymes and signaling cascades. Besides, copper serves as a crucial cofactor for several metalloenzymes involved in cancer metastasis, including SOD1, TGF-β, and LOX. LOX is an extracellular enzyme dependent on copper that is responsible for cross-linking collagen and elastin in the extracellular matrix (ECM) and plays a pivotal role in ECM reorganization during metastasis (Erez, 2015). Studies have demonstrated that copper can initiate cancer metastasis through the ATOX-ATP7A-LOX signaling pathway. When combined with ATOX1, copper can enhance tumor cell proliferation by upregulating Cyclin D1 expression (Jiang et al., 2023). Conversely, silencing ATOX1 or inhibiting ATP7A activity can reduce LOX function and inhibit tumor cell proliferation and migration (Shanbhag et al., 2019). Another copper-dependent enzyme called AOC1 has been shown to promote the proliferation and migration of colorectal cancer cells in both *in vivo* and *in vitro* experiments, exhibiting a significant correlation with unfavorable clinical outcomes among colorectal cancer patients (Liu et al., 2021).

Angiogenesis is an indispensable process in tumor progression, involving diverse stages such as endothelial cell proliferation and migration, vascular luminal formation, and vascular network construction (Cao et al., 2023). Copper not only regulates the release of angiogenic factors like fibroblast growth factor (FGF) and inflammatory cytokine IL-1α (Chen Z. et al., 2023), but also promote tumor angiogenesis and metastasis by directly activating various proangiogenic molecules, including VEGF, SOD1, FGF2, TNF-α, and IL-6 (Wang X. et al., 2023). For example, copper ions infiltrating the nucleus via CCS can enhance the expression and increase the persistence of hypoxia-inducible factor 1 (HIF-1), thereby stimulating VEGF expression and inducing tumor angiogenesis (Chen X. et al., 2023). Moreover, Cu can also regulate the affinity of angiopoietin to endothelial cells by directly binding to angiogenic factors (Wang Z. et al., 2023). In conclusion, copper’s pivotal role in tumor angiogenesis will further promote colorectal cancer progression and metastasis.

## 3 Cuproptosis: cell death induced by copper in colorectal cancer

**FIGURE 1:**
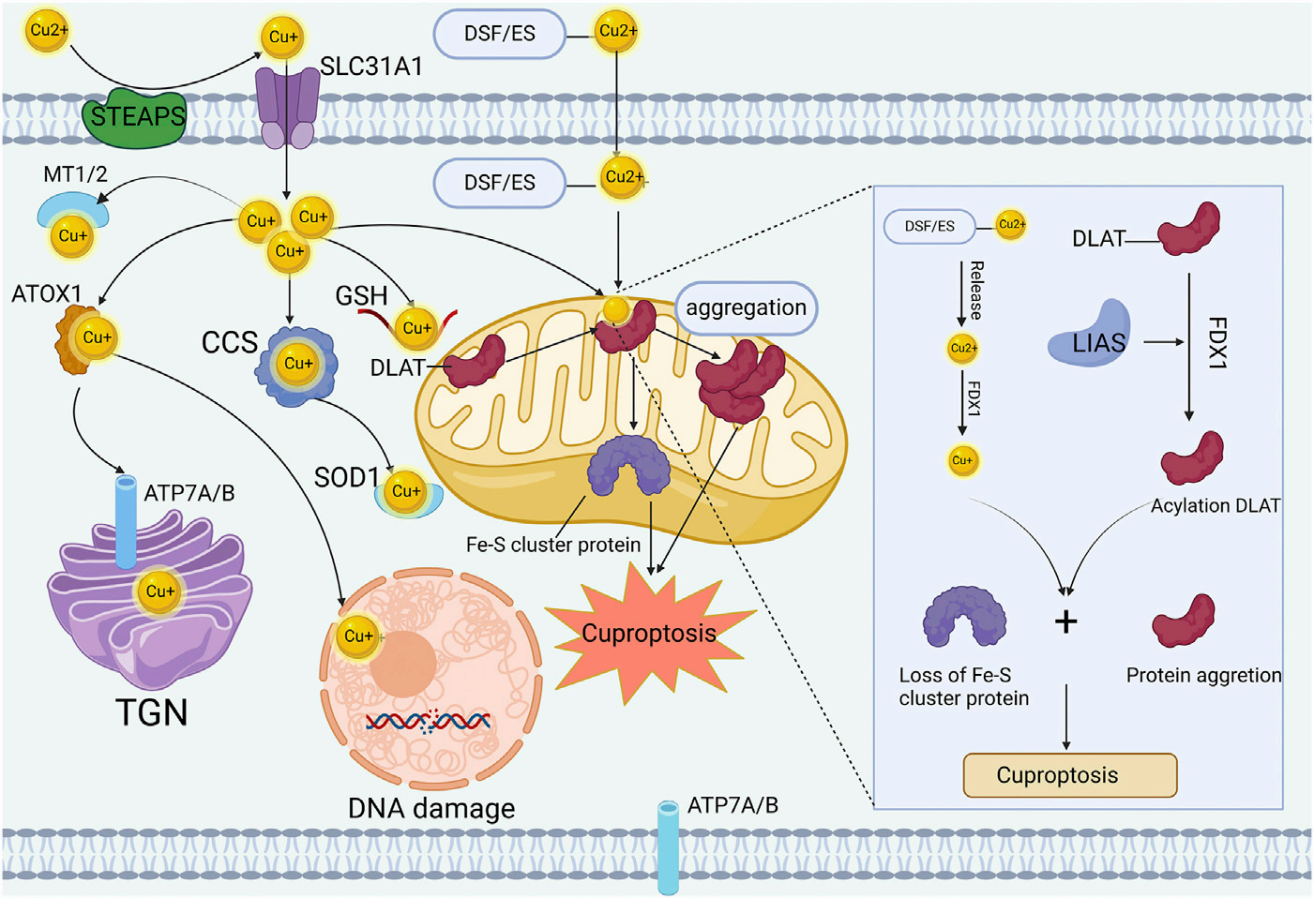
Mechanisms of cuproptosis. STEAP and DCYTB facilitate copper transitions to monovalent states for cellular entry through CTR1 in intestinal epithelial cells. Within these cells, copper associates with copper chaperones such as ATOX1, CCS, and SOD1, localizing to distinct subcellular sites like mitochondria, TGN, and nucleus. The copper ionophore DSF or ES facilitate the import of excess copper into the cell and further shuttle it to the mitochondria. Intensive copper binding to mitochondrial lipoylated TCA components triggers oligomerization of lipoylated proteins like DLAT and destabilizes Fe-S cluster proteins, inducing proteotoxic stress leading to cell death.

In 1978, Chan et al. reported that high concentrations of copper could induce fibroblast cell death (Chan et al., 1978). However, the underlying mechanism remains poorly understood. The conventional belief suggests that copper ions can enhance cell death by triggering the accumulation of reactive oxygen species (ROS), inhibiting proteasome activity, and causing mitochondrial dysfunction (Hu et al., 2022). Nevertheless, recent research has revealed cuproptosis as a novel form of regulated cell death, shedding light on the enigmatic mechanism behind copper-induced cell death. Copper-induced cell death is mediated through protein lipoylation. Excessive copper transfer to mitochondria leads to the reduction of Cu^2+^ to Cu^+^ by FDX1. Unlike other known forms of regulated cell death, elevated levels of Cu^+^ directly interact with dihydrolipoamide-acetyltransferase (DLAT), a lipoacylating component in the TCA cycle within mitochondria. This interaction results in oligomerization of lipoacylated DLAT protein and destabilization of Fe-S proteins, ultimately leading to proteotoxic stress and subsequent cellular death (Figure 1) (Tsvetkov et al., 2022). The recognition of cuproptosis highlights the crucial role of copper in tumor pathogenesis and emphasizes the potential efficacy of copper-based nanomedicines in colorectal cancer immunotherapy.

## 4 Cuproptosis and colorectal cancer TME

### 4.1 Colorectal cancer: immunosuppressive tumor microenvironment

The intricate tumor microenvironment consists of tumor cells, non-tumor cells (such as fibroblasts, endothelial cells, and immune cells), and non-cellular components including the extracellular matrix, cytokines, and growth factors (Wang et al., 2023c). The composition and infiltrative status of immune cells within TME are critical factors that influence tumor progression, prognosis, and treatment strategies (Guo et al., 2020). Based on their distinct functionalities, immune cells can be categorized as innate and adaptive immune cells. Innate immune cells encompass macrophages, NK cells, and MDSCs which constitute the body’s initial defense against foreign aggressors (Sadeghi et al., 2024). Tumor-associated macrophages (TAMs) can be classified into M1 and M2 profiles. M1 macrophages stimulate antitumor responses by producing proinflammatory cytokines such as IL-6, IL-12, IL-23, etc., whereas M2 macrophages promote tumor immune evasion by recruiting Tregs and secreting immunosuppressive cytokines like IL-10 and TGF-β (Prakash et al., 2023). As tumor progression occurs over time, M1 macrophages could gradually polarize into M2 macrophages (Sharifi et al., 2019). NK cells possess potent tumoricidal capabilities. Insufficient infiltration of NK cell correlates with adverse cancer progression and prognostic indices (Wang et al., 2021). The reduced levels of infiltrating NK cell in CRC tissues along with compromised cytotoxic activity may be associated with the establishment of an immunosuppressive TME in CRC (Halama et al., 2011). MDSCs represent a diverse group of regulatory cells derived from the myeloid lineage. Research indicates that MDSCs exhibit elevated levels in CRC patients’ circulation, which are associated with diminished antitumor immunity levels along with advanced tumor stage and metastasis (Umansky et al., 2016). Adaptive immune cells comprise T cells and B lymphocytes, which elicit a sustained antitumor immune response (Gajewski et al., 2013). T cells, the predominant immune cell population in colorectal cancer, can be categorized as CD8^+^ T cells and CD4^+^ T cells. CD8^+^ T cells produce cytotoxic enzymes such as granzyme B and perforin, and also exert antitumor activity by generating the proinflammatory signaling mediator TNF-α (Guo et al., 2020). Similar to CD8^+^ T cells, CD4^+^ T cells exhibit cytotoxic effects through the Fas/FasL and GZMB/Perforin pathways (Russell and Ley, 2002). B lymphocytes are responsible for antibody production. However, their role in the TME remains controversial. A recent study has suggested that B lymphocytes may play a vital role in the therapeutic response to ICIs by modulating T cell activation and functionality (Helmink et al., 2020). Regulatory T cells, a subset of CD4^+^ T lymphocytes, could produce IL-10 through multiple pathways to suppress the cytotoxic activity of both CD8^+^ and CD4^+^ T cells and downregulate the expression of IFN-γ and TNF-α. Consequently, their accumulation within tumors hinders the expansion of anti-tumor effector T cells and promotes an immunosuppressive microenvironment (Oshi et al., 2020).

### 4.2 Copper dyshomeostasis and the tumor microenvironment in colorectal cancer

**FIGURE 2:**
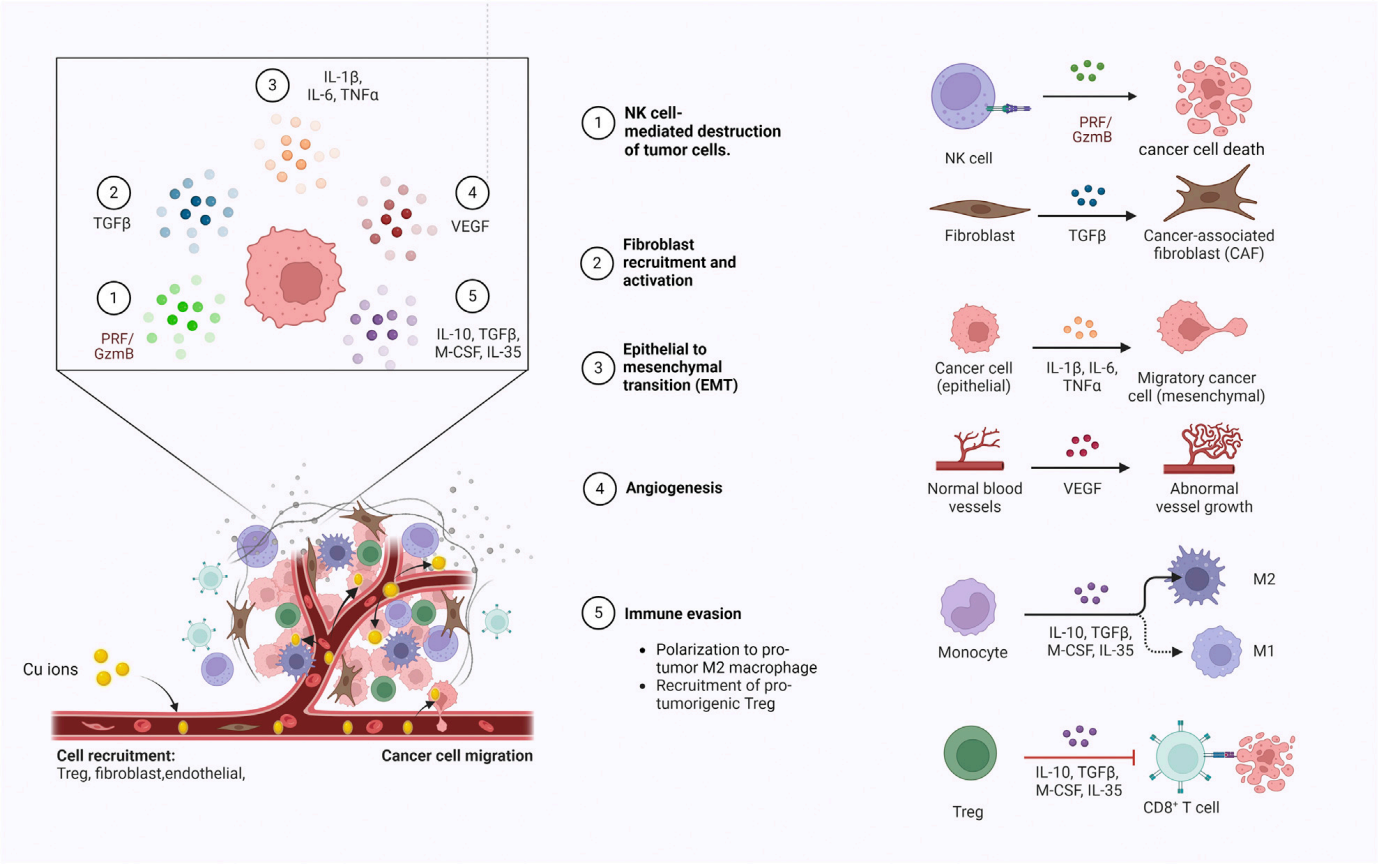
Copper ions enter the tumor microenvironment via blood circulation to modulate the migration of immune cells, the release of immune effector molecules and tumor angiogenesis.

Copper is an essential trace element in the human body, playing a key role in maintaining immune homeostasis and enhancing immune system function (Figure 2), and potentially influencing the development and progression of colorectal cancer through immune-related mechanisms (Mo et al., 2024). The dyshomeostasis of copper can potentially influence the functionality of the immune system through diverse mechanisms, which subsequently impacts the initiation of anti-tumor immune responses and the efficacy of immunotherapy. Primarily, copper is crucial for keeping the normal activity of immune cells, and assists in the synthesis of cytokines and chemokines, which play an integral role in managing the body’s immune response (Ladomersky and Petris, 2015). A disruption of copper homeostasis in the body may result in compromised production of immune-related molecules, reducing the capacity of the immune system to monitor and eliminate cancer cells. Research has demonstrated that apart from a reduction in the count of circulating neutrophils in patients with copper deficiency, the functionality of neutrophils to generate immunoactive substances such as superoxide anions is also diminished (Babu and Failla, 1990). Furthermore, copper deficiency also brings about a decrease in the count of antibody-producing cells within the body (Prohaska and Lukasewycz, 1981). Besides, studies have shown that copper deficiency could also impairs the number and functionality of cytotoxic T cells and helper T cells, which ultimately limits the secretion of immune effectors and leading to immunosuppression (Cheng et al., 2022). Maintaining intracellular and extracellular copper homeostasis is a premise for proper macrophage function. Copper deficiency can lead to macrophage depletion (Bala et al., 1991), while excessive copper can impair macrophage function (Zhao and Zhao, 2019). Additionally, copper ions have been shown to induce the polarization of M1 macrophages (Díez-Tercero et al., 2022). Furthermore, copper also exhibits regulatory effects on the expression of the immune checkpoint PD-L1. Voli’s study have demonstrated that copper deficiency can reduce PD-L1 mRNA translation by suppressing the JAK/STAT pathway and can also decrease PD-L1 expression by promoting its ubiquitination and degradation in tumor cells (Voli et al., 2020).

Conversely, intracellular copper overload can lead to cell death. Copper-induced cell death is a new type of regulatory cell death that occurs when excess copper in the cell binds directly to the lipoacylated component of the tricarboxylic acid cycle, known as cuproptosis. Studies have shown that cuproptosis can modulate the efficacy of immunotherapy by affecting the infiltration of immune cells in the tumor microenvironment. For example, Zeng et al. found that cuproptosis in microsatellite-stabilized colorectal cancer cells can enhance the cytotoxicity of CD8^+^ T cells by downregulating the WNT signaling pathway (Zeng et al., 2024).In tumor cells, cuproptosis also can improve anti-tumor immune responses through cGAS-STING signaling. The cGAS-STING pathway is an essential innate immune signal that plays a crucial role in the anti-tumor immune response. Jiang et al. found that, under the induction of ES and CuCl_2_, the cGAS-STING activity in dendritic cells co-cultured with cuproptosis-activated ccRCC cells increased in a dose-dependent manner, and the combination of cuproptosis inducers (ES and CuCl_2_) and anti-PD-1 therapy can synergistically increase the levels of circulating CD45^+^, CD8^+^ T lymphocytes, enhance the pro-inflammatory response and the production of type I IFN, thereby amplifying the anti-tumor immune response and enhancing the efficacy of anti-PD-1 therapy (Jiang et al., 2022). However, further investigation is needed to fully understand the impact of copper on the human immune system.

### 4.3 Key cuproptosis genes and the tumor microenvironment in colorectal cancer

Key cuproptosis genes include FDX1, SLC31A1, DLAT, CDKN2A, and LIAS, among others. Multiple bioinformatics investigations have demonstrated the intricate association of these genes with immune-related gene expression and infiltration of immune cells in the TME. This suggests their potential as promising targets and biomarkers for enhancing the effectiveness of immunotherapy in colorectal cancer (Zhu et al., 2022).

The iron-sulfur protein FDX1, an upstream modulator of protein lipoacylation, plays a significant role in promoting the synthesis of the key enzyme LIAS during DLAT lipoacylation (Dreishpoon et al., 2023). Studies have demonstrated a positive correlation between FDX1 expression and infiltrated levels of CD8^+^ T cells, NK cells, and neutrophils, while showing a negative correlation with infiltrated levels of CD4^+^ T cells and cancer-associated fibroblasts. These findings suggest that elevated FDX1 expression is beneficial in preventing tumor immune evasion (Wang L. et al., 2023). Furthermore, FDX1 expression is significantly associated with various immune-related genes including chemokines, chemokine receptors, MHC molecules, and immune checkpoint proteins (Yang L. et al., 2022). Taken together, these observations indicate that regulating the FDX1 expression may hold promise for enhancing immunotherapy efficiency in colorectal cancer. CTR1, also known as SLC31A1, is a copper transporter protein that plays a crucial role in maintaining intracellular copper homeostasis. (Tury et al., 2023). Elevated levels of intracellular copper induced by SLC31A1 can activate molecular cascades such as NFκB, JAK/STAT, and PI3K/AKT/mTOR to upregulate PD-L1 expression in tumor cells (Voli et al., 2020). Therefore, targeting SLC31A1 in combination with anti-PD-L1 therapy may be a promising approach for cancer treatment. DLAT is an inner mitochondrial membrane protein that regulates tumor progression through pyruvate oxidation, citric acid cycle and glycolysis processes. (Ganetzky et al., 2024). Studies have shown that DLAT can shape the immunoreactive TME by increasing cytotoxic T cell levels and reducing T cell depletion. Moreover, DLAT expression positively correlates with the infiltration levels of CD8^+^ T cells, neutrophils, macrophages B cells and DC cells in CRC patients (Chu et al., 2023). Thus, DLAT could potentially enhance the response of CRC to immunotherapy by shaping “hot” TMEs.

Single sample gene set enrichment analysis was utilized by Qing et al. in their investigation to calculate individual cuproptosis scores (CS), which exhibited a significant positive correlation with the expression of most cuproptosis-related genes, indicating that CS can serve as an indicator of the cuproptosis status. Their findings demonstrated that CS levels were substantially higher in tumor tissue compared to healthy tissue, and there was a strong positive association between CS and resting mast cells, eosinophils, activated CD4^+^ T cells, and dendritic cells. Additionally, CS showed a marked negative correlation with Tregs, NK cells, M0 macrophages, follicular helper T cells, and memory B cells (Qin et al., 2023). Therefore, these cuproptosis-related genes have the potential to be novel immunotherapeutic biomarkers.

### 4.4 Cuproptosis-related lncRNAs and the tumor microenvironment in colorectal cancer

Long non-coding RNAs (lncRNAs) play significant roles in various stages of tumor progression, including proliferation, apoptosis, angiogenesis, and metastasis. They significantly contribute to tumorigenesis, development, dissemination, and metastasis (Coan et al., 2024). Moreover, lncRNAs can regulate mRNA translation in a base-pair-dependent manner and modulate signaling pathway such as WNT/β-catenin, PI3K/Akt, mTOR, and TP53 through interactions with diverse proteins and lipids (Galamb et al., 2019). Importantly, several studies have demonstrated the critical functions of lncRNAs in controlling colorectal cancer cell proliferation, apoptosis, epithelial-mesenchymal transition (EMT), invasion, and drug susceptibility via gene transcriptional regulation and post-transcriptional modulation (Chen and Shen, 2020). Therefore, lncRNAs hold great potential as biomarkers for early identification, diagnosis, and prognosis prediction of colorectal cancer. Additionally, the therapeutic strategies targeting cuproptosis-related lncRNAs (CRLs) are expected to provide novel insights for cancer treatment.

Yang et al. (2023) developed a risk-scoring model incorporating six cuproptosis-related lncRNAs (AC009315.1, PLS3-AS1, ZEB1-AS1, AC007608.3, AC010789.2, and AC010207.1) that exhibit strong correlations with colorectal cancer prognosis. They observed reduced immune score and CD8^+^ T cell infiltration in the high-risk group, while the low-risk group showed lower immune rejection, immune dysfunction, and TIDE scores compared to the high-risk group. Moreover, these lncRNAs included in the risk model demonstrated positive associations with several immune checkpoint proteins such as PD-L1 and CTLA4, suggesting their potential as biomarkers for prognostication and prediction of immunotherapy response in CRC patients. Similarly, Pang et al. utilizing CRLs including SNGH16, LINC02257, PRARP-AS1,and LENG8-AS1 in their risk model, revealed significantly higher infiltration of naïve B cells,CD8^+^ T cells, follicular helper T cells,M1 macrophages, and resting mast cells in the low-risk group compared to the high-risk group (Pang et al., 2023). This may be attributed to CRL’s influence on the immune status of CRC patients. These findings provide novel insights into CRC prognosis research and contribute to advancements in CRC immunotherapies.

## 5 Crosstalk between cuproptosis and ICD

Cuproptosis and immunogenic cell death (ICD) are two essential forms of programmed cell death that play significant roles in various physiological and pathological processes, including cancer. ICD is a regulated form of cell death that triggers an immune response against dying cancer cells’ antigens, thereby preventing tumor recurrence and metastasis (Pan et al., 2024). Upon induction of ICD, a series of signaling molecules known as damage-associated molecular patterns (DAMPs), such as extracellular ATP, surface-exposed calreticulin, and released high-mobility group box 1 protein (HMGB1), among others, are generated (De Silva et al., 2024). These DAMPs bind to pattern recognition receptors (PRRs) present on the surface of dendritic cells (DCs), initiating a cascade of cellular responses and potentially amplifying innate and adaptive immune responses (Yatim et al., 2017). Consequently, activated T cells infiltrate multiple tumor sites, facilitating the elimination of cancer cells while also enhancing the tumor antigen-specific T-cell immune response through the release of tumor-associated antigens from dead cancer cells (Deng et al., 2024). Recent studies have shown that treatment protocols involving cuproptosis-inducing drugs can effectively stimulate ICD, suggesting a potential correlation between cuproptosis and immunogenic cell death (Li et al., 2023a). For example, Kaur demonstrated that the reticulum-targeting Copper (II) complex can increase intracellular ROS levels, induce ER stress, stimulate the release of damage-related molecular patterns, and ultimately promote immunogenic cell death in breast cancer stem cells (Kaur et al., 2020). Furthermore, Cuproptosis is expected to enhance ICD and initiate a robust anti-tumor immune response within the tumor tissue, making it a promising modality for immunotherapy against malignant tumors (Liang et al., 2024). Zheng et al. reported that DSF/Cu co-delivery system can trigger tumor cell autophagy and induce immunogenic cell death, promote M1 macrophage polarization and dendritic cell maturation, and enhance the killing effect of CD8^+^ T cells and NK cells, thereby promoting anti-tumor immune response and tumor regression (Zheng et al., 2020). Zhao et al. also found that DSF/Cu can induce ICD in OSCC cells and promote the maturation and activation of DCs, thereby improving the anti-tumor immune response (Zhao et al., 2024). Hence, cuproptosis inducers are expected to enhance the efficacy of immunotherapy by promoting ICD, forming a promising combination synergistic strategy for colorectal cancer immunotherapy. However, further research is needed to explore the intrinsic association between cuproptosis and the mechanism underlying immunogenic cell death.

## 6 Enhancing the efficacy of immunotherapy against colorectal cancer through the utilization of cuproptosis-inducing nanomedicines

In recent years, the emergence of cuproptosis has introduced a novel strategy for colorectal cancer treatment, while nanotechnology’s progression exhibits potential for broad pharmaceutical applications. Nanomedicines, distinct from conventional antitumor drugs, exhibit benefits including small size, stable structure, high permeation, robust controlled release capacity, and favorable biocompatibility. Moreover, nanomedicines can specifically target tumors via acoustic, optical, or thermal responses, enhancing synergies with therapeutic strategies like photodynamic therapy, chemodynamic therapy, and sonodynamic therapy, forming sophisticated drug delivery systems with pronounced clinical transformation potential. Consequently, the development of innovative copper-based nanomedicines to induce cuproptosis of colorectal cancer cells may evolve into an extremely promising tumor therapeutic strategy.

Immunotherapy is a novel clinical strategy for treating tumors by stimulating the body’s innate immune system to recognize and eliminate tumor cells (Demaria et al., 2019). Currently, various types of immunotherapies have been developed, including immune checkpoint inhibitors, tumor vaccines, chimeric antigen receptor T cells (CAR-T), et al. (Liu Y. et al., 2023). The significant impact of copper and cuproptosis on the tumor microenvironment highlights the potential to bolster cuproptosis induction in combination with immunotherapy as a novel research avenue for enhancing colorectal cancer immunotherapy. For instance, Huang et al. synthesized a copper-based nanoplatform BSO-CAT@MOF-199@DDM (BCMD). The release of Cu^+^ during degradation in the acidic tumor microenvironment can not only induce cuproptosis but also stimulate dendritic cell activation and cytotoxic T cell infiltration, thereby reshaping the immunosuppressive TME. Furthermore, BSO-CAT@MOF-199@DDM (BCMD) has shown significant potential to enhance the efficacy of anti-tumor immunotherapy when combined with the immune checkpoint inhibitor α-PD-L1 (Huang et al., 2023).

**FIGURE 3:**
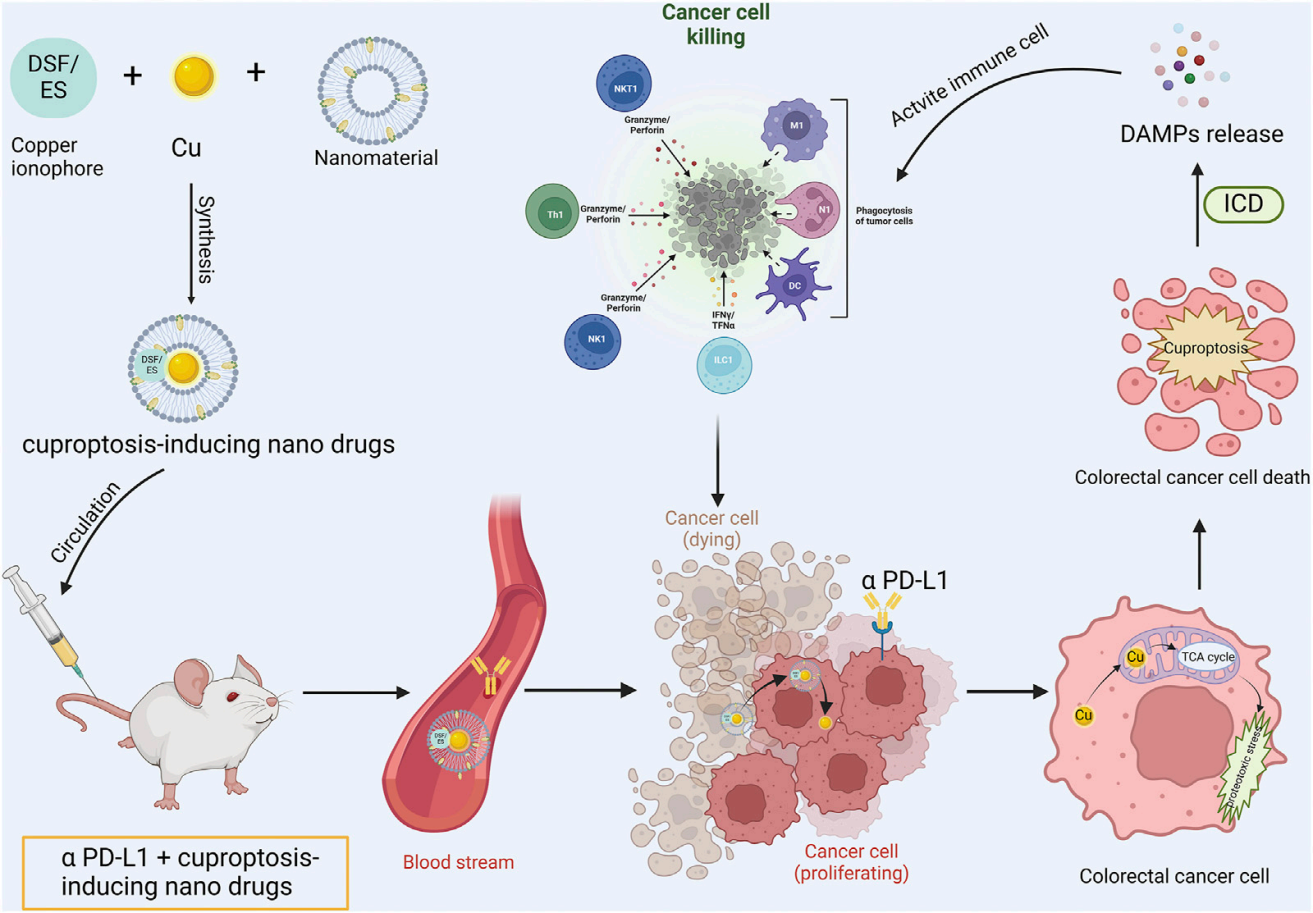
Cuproptosis-based nanomedicine synergizing with ICIs operational mode diagram. The copper ionophore is co-encapsulated with copper and nanomaterials into minute particles of nanomedicine. Subsequently, the engineered cuproptosis-induction nanomedicine is co-administered into the body in conjunction with anti-PD-L1 antibody. The copper-bearing nanomedicine is internalized by tumor cells and liberates copper ions. Copper ions penetrate mitochondria and bind with lipoylated proteins to induce tumor cell cuproptosis. Concurrently, the activation of ICD prompts the release of DAMPs, reverse the immunosuppressive tumor microenvironment and activate the anti-tumor immune response, thereby exhibiting synergism with anti-PD-L1 antibody.

Potential copper-related drugs, such as copper ionophores, hold considerable potential in colorectal cancer treatment when integrated with advanced nanomaterials and synergized with established therapeutic methods (including chemotherapy, radiotherapy, immunotherapy, etc.). Currently, nanomaterials employed in copper-based nanomedicines comprise polymer nanoparticles (PNPs), liposomes, polymer micelles (PM), Mxenes, Cu-MOF, hydrogels, etc. PNPs are preferred for their versatility, biocompatibility, controlled release, biodegradability, and adjustable size, shape, and surface charge. Moreover, through surface modification, PNPs can be precisely directed to specific body tissues to enhance drug efficacy (Beach et al., 2024). Nevertheless, the clinical application of PNPs necessitates further exploration, encompassing solvent toxicity during preparation, acidic degradation by-products, biphasic drug release, and mass production challenges. Liposomes, which are composed of phospholipid bilayers, offer biocompatibility, biodegradability, high drug loading rate and encapsulation of both hydrophilic and hydrophobic substances. They can extend drug circulation half-life and generate high local drug concentrations in tumors (Zununi Vahed et al., 2017). However, the incorporation of water-soluble inorganic copper ions may compromise liposome structural stability. Polymeric micelles, possessing superior biocompatibility, active targeting, stimulatory reactivity, and minimal toxicity to healthy cells, can dissolve multiple drugs within the micellar core. Their small size facilitates their accumulation in the TME via enhanced permeability and retention effects (Hari et al., 2023). Nonetheless, PM’s stability may result in slow drug release, decreasing it’s therapeutic efficacy. MXenes, a unique two-dimensional material, offers facile production and functionalization, large surface area, biocompatibility, and ideal mechanical properties, along with excellent photothermal and photodynamic effects, thus enabling effective non-invasive anti-cancer therapy (Fadahunsi et al., 2022). However, its stability, controlled drug release, and biodegradability remain issues. MOF is a crystalline particle filled with molecular-sized pores formed by metal ions and organic linkers, possesses high porosity, large surface area, excellent loading capacity, and biodegradability. It serves as a promising platform for nano-drug delivery and tumor treatment. Cu-MOF combines diverse therapeutic potentials of copper ions, including driving Fenton-like reactions, inducing CDT, and generating ROS as a photosensitizer for PDT. Additionally, the high specific surface area and porosity of Cu-MOF make it an effective carrier for chemotherapeutic drugs and photosensitizers (Song et al., 2024). Hydrogels, due to their hydrophilicity, biodegradability, extensive swelling ability, and suitable mechanical properties, are promising materials in cancer therapy. Their injectability, self-repair, controllable drug release, and excellent biocompatibility make hydrogels widely utilized in cancer treatment (Farasatkia et al., 2024). In conclusion, considering the intrinsic characteristics of different nanomaterials and integrating them organically with copper-based drugs will help developing more potent nanoplatforms for inducing colorectal cancer cuproptosis and immunotherapy synergism (Figure 3).

### 6.1 Nano-encapsulated copper ionophore

Excessive intracellular copper can be directed towards the mitochondria, where its interaction with fatty acylated proteins initiates proteotoxic stress, ultimately leading to cancer cell death through a process known as cuproptosis (Tsvetkov et al., 2022). Copper ion carriers, such as DSF, can form a neutral lipophilic complex with copper, enhancing the accumulation of copper within the cell (Allensworth et al., 2015), which suggests that utilizing copper ionophores may be a promising approach for inducing cancer cell cuproptosis in cancer therapy. However, current copper ionophores have demonstrated limited therapeutic efficacy either independently or in combination with other drugs (Kang et al., 2023). This may be due to an active balance regulatory mechanism present in most cancer cells that controls the cellular content of copper and prevents damage from excessive influx of copper ions over a brief period. Additionally, systemic administration of copper ionophore is associated with issues such as insufficient dosages and severe adverse reactions (Pan et al., 2022). Therefore, there is an urgent need to develop more effective and precise delivery systems for these agents. Nano-drug delivery systems offer targeted accumulation at tumor sites and synergistic effects when combined with other therapies to enhance treatment outcomes. Thus, integrating nanomaterials with copper ionophore could lead to innovative drug delivery systems that significantly improve colorectal cancer immunotherapy.

Elesclomol (ES), a copper ionophore primarily targeting mitochondrial metabolism, was initially developed as a chemotherapy adjuvant and can induce copper-dependent mitochondrial apoptosis to exhibit anti-cancer activity (Gao et al., 2023). However, due to the rapid clearance and metabolism of ES in the circulation, short half-life and limited tumor accumulation, its anti-cancer efficacy in clinical trials is suboptimal (O'Day et al., 2013). Recently, the integration of nanomedicine with elesclomol has facilitated the application of advanced nanotechnology to manipulate the pharmacological properties of ES and synergize with immunotherapy. Copper oxide nanoparticles (CuO NPs), possessing exceptional attributes like efficient drug loading and biological compatibility, serve ideally as metal oxide nanoparticle-derived carriers for inducing cuproptosis. Lu et al. integrated the copper ionophore ES with CuO NPs within a polyethylene glycol polymer to form the cuproptosis-stimulating nanosystem ES@CuO. Upon ingestion by tumor cells, ES@CuO degrades and releases Cu^2+^ and ES, thereby increasing intracellular copper levels and triggering cuproptosis, significantly suppressing tumor growth. Furthermore, ES@CuO can stimulate immune responses via increased tumor-infiltrating T lymphocytes and inflammatory cytokine secretion, thereby significantly enhancing the anti-tumor efficacy of anti-PD-1 antibodies in mouse melanoma (Lu X. et al., 2024). Guo et al. synthesized a ROS-responsive biodegradable polymer PHPM and utilized it to encapsulate ES and Cu into nanoparticle NP@ESCu. Upon internalization by tumor cells, excessive intracellular ROS triggers targeted release and accumulation of ES and Cu within tumor cells. The synergistic effect between ES and Cu not only evokes cancer cell cuproptosis leading to DAMPs release that facilitates DC cell maturation and augments CD8^+^ T cell infiltration but also transforms immune “cold tumors” into “hot tumors.” Additionally, NP@ESCu notably enhances PD-L1 expression in tumor cells, remarkably improving the response rate of anti-PD-L1 therapy, thus enabling synergy between cuproptosis induction and immunotherapy for an enhanced anti-tumor effect (Guo B. et al., 2023). The combination of NP@ESCu with anti-PD-L1 therapy provides both a novel therapeutic strategy for colorectal cancer as well as serves as a model for nano-drugs fused with cuproptosis to enhance the efficacy of tumor immunotherapy.

DSF represents another extensively studied copper ionophore, and its main metabolite diethyldithiocarbamate, can form CuETs by binding with Cu^2+^ to exhibit anti-tumor efficacy (Lu et al., 2022). Recent studies have demonstrated that DSF has the ability to modulate the tumor immune microenvironment through manipulation of tumor-associated macrophages and regulation of PD-L1 expression, showing significant potential for synergistic immunotherapy in colorectal cancer. For instance, Zeng et al. discovered that disulfiram enhances the anti-PD-1 treatment efficacy in the 4T1 breast cancer mouse model by stimulating IRF7 to elevate PD-L1 expression in TNBC cells and augmenting the infiltration of CD8+T cells (Zheng et al., 2021). However, DSF lacks sufficient tumor targeting capacity and is limited by its poor water solubility and systemic toxicity caused by exogenous Cu^2+^, which hinders its practical application. To overcome these challenges, nanomedicines with specificity towards the tumor microenvironment are promising solutions. Li et al. developed PH-responsive lipid-coated calcium phosphate nanoparticles loaded with Cu^2+^ and DSF (Cu-LCP/DSF NPs), which release Cu^2+^ and DSF upon degradation in an acidic tumor microenvironment, leading to the generation of cytotoxic metabolite CuET. In addition to direct cytotoxicity, CuETs can induce immunogenic cell death of cancer cells effectively, rectify the immunosuppressive TME, thereby enhancing the efficacy of immune checkpoint inhibitors (Li Q. et al., 2022). This pH-responsive nanoparticle exhibited excellent safety profile and remarkable anti-tumor activities in CT26 mouse model, providing valuable insights for developing innovative nanomedicines to augment immunotherapy for colorectal cancer. In another investigation, Liu et al. developed a biomimetic system called CuX-P by coating DSF/Cu incorporated MXene nanosheets onto T cell membranes that overexpress PD-1. This system demonstrated the ability to identify and adhere to tumor cells’ PD-L1, exhibiting remarkable tumor-targeting capability. Upon internalization by tumor cells, CuX-P induced cuproptosis through the liberation of DSF/Cu^2+^, while also triggering elevated CRT expression and HMGB1 release, thereby facilitating the recognition and activation of tumor-associated antigens by dendritic cells. Additionally, DSF/Cu escalated the expression of PD-L1 in tumor cells (Liu T. et al., 2023). Thus, CuX-P can evocate a potent anti-tumor immune response while inducing cuproptosis, which considerably enhances the efficacy of anti-tumor immunotherapy.

Chimeric antigen receptor T cells (CAR-T) represent a groundbreaking immunotherapy approach that has demonstrated significant success in the treatment of hematological malignancies. However, their effectiveness in treating solid tumors is currently limited (Neeser et al., 2023). A recent study conducted by Wang and his team explored a potential solution by examining the effect of radiation therapy combined with DSF/Cu in triggering cytotoxic stress responses in cancer cells. This technique can significantly increase immunogenic cell death of differentiated cancer cells and CSCs while promoting CAR-T cell proliferation and inducing differentiation of effector memory T cells, thereby substantially inhibiting metastatic tumor progression (Wang et al., 2023e).

### 6.2 Sonodynamic therapy combined with nanomaterials

Sonodynamic therapy (SDT), a non-invasive ultrasound modality, holds significant potential for precise targeting and eradication of solid tumors due to its exceptional tissue penetration and efficacy in deep tumor ablation (Wang et al., 2022). However, the therapeutic effectiveness of SDT is limited under hypoxic tumor microenvironment conditions due to reduced production of ROS (Yang Z. et al., 2022). To address this issue, Chen et al. developed a biomimetic nanorobot (SonoCu) enclosed within a macrophage membrane to facilitate synergistic anticancer effects through cuproptosis and SDT. SonoCu exhibits remarkable tumor targeting ability, tissue penetration, internalization into cancer cells, and can specifically induce ultrasound-responsive cytotoxicity towards cancer cells while minimizing adverse reactions. Furthermore, SonoCu-mediated cuproptosis enhances the destructive impact of SDT on cancer cells by promoting ROS accumulation and protein toxic stress mechanisms, thereby presenting a novel strategy for augmenting sonodynamic therapy and stimulating cancer cell cuproptosis that may have synergistic implications in colorectal cancer immunotherapy (Chen K. et al., 2023). Similarly, Yu et al. engineered a sonar-activated semiconductor polymer nanoreactor (SPNLCu). The sonar-activated LOx cascade release efficiently depletes lactate in TME and mitigates its immunosuppressive effects. Furthermore, Cu^2+^ mediates cupptosis and induces immunogenic cell death, thus potentiating anti-tumor immune responses, demonstrating promising efficacy in mouse pancreatic cancer models (Yu N. et al., 2024). Concurrently, Tang et al. synthesized Cu-substituted ZnAl ternary LDH nanosheets (ZCA NSs), a multifunctional platform for synergistic SDT/Cuproptosis combination therapy. The Jahn-Teller effect engendered by Cu^2+^ optimizes the electronic structure of ZCA NSs, enhancing sonodynamic processes. ZCA NSs augment SDT efficiency by depleting endogenous GSH to amplify oxidative stress. Additionally, the resultant excess Cu^2+^ triggers cuproptosis, while cytotoxic ROS foster dendritic cell maturation, stimulating systemic antitumor immunity, and ultimately hindering distal metastasis in mouse CT26 colon cancer models (Tang et al., 2024).

### 6.3 Photosensitive nano biomaterials to induce cuproptosis: PTT/PDT therapy

Photothermal therapy (PTT) induces the anti-tumor immune response by heating and eliminating tumors, which leads to the production of tumor antigens and other immune signals. This process activates the body’s immune system to target and attack the tumor cells. Various photothermal agents, such as gold NPs and CuS NPs, have been developed for cancer treatment (Li et al., 2020). When these photothermal agents accumulate at the tumor site, localized hyperthermia can be induced by laser irradiation to enhance immunogenic cell death, promote the release of DAMPs, and further eliminate tumors by presenting tumor-associated antigens, facilitating dendritic cell maturation and activating cytotoxic T lymphocytes (Deng et al., 2019). For example, CuMoO_4_ nanodots fabricated by Zhang et al. not only efficiently induce cuproptosis in tumor cells but also exhibit excellent photothermal conversion efficiency under near-infrared II (NIR-II) laser irradiation. Furthermore, CuMoO_4_ nanodots with multi-enzyme activity can also modulate the tumor microenvironment, leading to immunogenic cell death and activation of the immune response, providing a promising nanoplatform for multimodal combination therapy of tumors (Zhang et al., 2023). Similarly, Hu et al. developed a mesoporous polyamine-based nanoplatform (MPDA-Cu/DQ-GOx-FA/PEG, MCDGF), which not only offers superior photothermal therapeutic effect through nanocarrier mesoporous polydopamine but also induces Cu(DTC)_2_-mediated immunogenic cell death, enhances macrophage polarization towards anti-tumor subtypes, amplifies CTL-mediated anti-tumor immune response, and thus presents a new strategy for synergistic cancer treatment integrating PTT, chemotherapy, and immunotherapy (Hu et al., 2023). In another investigation, Ruan et al. synthesized a microbial nanohybrid E.coli@Cu_2_O composed of *E. coli* and Cu_2_O nanoparticles, which can specifically accumulate at colorectal tumor sites after intravenous administration. In response to elevated levels of H_2_S in colorectal tumors, Cu_2_O can be converted into CuS, demonstrating a strong photothermal effect under NIR-II laser irradiation. Furthermore, E.coli@Cu_2_O not only catalyzes the initiation of cuproptosis through DLAT oligomerization but also reverses the immunosuppressive tumor microenvironment in colorectal cancer by enhancing dendritic cell maturation and T cell activation, and synergizes with immune checkpoint inhibitors (Ruan et al., 2024). Studies have shown that abnormal glycolysis and high lactate levels in tumor cells may affect the sensitivity of tumor cells to cuproptosis, and elevated lactate levels can also form a highly immunosuppressive tumor microenvironment. Therefore, copper-human serum albumin nanocomplex loaded gold nanocages with bacterial membrane coating (BAu-CuNCs) were developed. Upon near-infrared laser exposure, copper-human serum albumin (Cu-HSA) release is amplified, copper ions are liberated via disulfide exchange reaction with intratumoral GSH, thereby triggering cuproptosis and initiating immunogenic cell death in tumors, reversing immunosuppressive TME. Simultaneously, under NIR illumination, gold nanocages (AuNCs) can stimulate the overproduction of ROS, thereby suppressing glycolysis, diminishing lactate and ATP levels, and ultimately augmenting tumor cell sensitivity to cuproptosis, and amplifying the anti-tumor immune response (Zafar et al., 2024). Furthermore, Wu et al. engineered a bioorthogonal Cu single atom nanozyme (CuSACO) loaded with high-efficiency catalase, oxidase, and peroxidase-like multi-enzyme activities, demonstrating superior photothermal performance upon 1064 nm laser irradiation. CuSACO effectively suppresses orthotopic breast tumor growth and metastasis by integrating the diverse effects of nanozymes with multi-enzyme catalytic activity, cuproptosis inducers, photothermal therapy, and immunotherapy. This discovery underscores the immense potential of innovative strategies utilizing multiple mechanisms to enhance the potency of cuproptosis-inducing nanomedicines (Wu L. et al., 2024).

Tumor vaccines have the potential to effectively stimulate the body’s anti-tumor immune response and eliminate tumor cells. However, current conventional tumor vaccines suffer from limitations such as reduced bioavailability, inadequate lymphatic reflux, and low antigen/adjuvant encapsulation rate, which hinders their long-term effectiveness and leads to unsatisfactory anti-tumor effects (Sun et al., 2024). To address these issues, Shen et al. developed a photothermally activated dendrimer nano-vaccine G5-PBA@CuS/antigen/cGAMP. This nano-system can integrate photothermal therapy to eliminate primary tumors upon laser irradiation. Additionally, the PBA fragment attached to the vaccine can capture the primary tumor antigen and serve as an *in situ* vaccine to initiate adaptive anti-tumor immune responses, effectively inhibiting the development of distal tumors (Shen S. et al., 2023). Furthermore, intracellular accumulation of copper may exert antitumor effects by inducing cuproptosis in cancer cells.

PDT (photodynamic therapy) is a therapeutic approach that utilizes photosensitizers, light, and oxygen to produce reactive oxygen species and leading to apoptosis (Yu L. et al., 2024). This technique is highly targeted, minimally invasive, and has minimal toxicity. In the study by Liang et al., they synthesized a copper synergistic nano-assembly (CCNAs) that incorporated photosensitizer-prechemotherapy and immune checkpoint inhibitors to induce cuproptosis and enhance cancer immune therapy. The ZnPc component of CCNAs exhibited photodynamic reactions under NIR-II laser irradiation, while the presence of Cu^2+^ not only enhanced the photodynamic effect by catalyzing ROS but also promoted the aggregation of lipoacylated proteins within mitochondria, inducing cuproptosis in tumor cells. PDT-induced tumor apoptosis and Cu-induced cuproptosis can enhance ICD, resulting in a strong anti-tumor immune response with the assistance of immune checkpoint inhibitors (Liang et al., 2024). Therefore, simultaneous administration of photosensitizers and Cu^2+^ via a nano-delivery system can serve as an effective strategy for synergistic tumor therapy, enhancing PDT-induced apoptosis and Cu^2+^-induced cuproptosis. In another study, Liu et al. designed an *in situ* therapeutic vaccine by fabricating a copper-tetrahydroxybenzoquinone (Cu-THBQ/AX) nano-MOF, utilizing the unique antigen of the primary tumor to stimulate a personalized, potent, and enduring anti-tumor immune response. Cu-THBQ/AX impairs ATP7A function, triggers copper ion accumulation, instigates tumor cell cuproptosis, and activates the *in situ* type I photodynamic action of Cu-THBQ/AX via alkaline phosphatase overexpressed on the tumor cell membrane, generating O_2−_ and ·OH, inducing caspase-3-mediated pro-inflammatory pyroptosis, thereby effectively transitioning the tumor microenvironment from a “cold” state to a “hot” state, suppressing tumor growth and metastasis. (Liu Y. et al., 2024). These works provide a valuable insight into the therapeutic management of colorectal cancer using cuproptosis combined with immunotherapy.

### 6.4 Chemodynamic therapy combined with nanomaterials

Chemodynamic therapy (CDT) primarily converts intracellular hydrogen peroxide into intensely toxic hydroxyl radicals via a Fenton or Fenton-like reaction, stimulating the production of endogenous ROS to catalyze tumor cell demise (Tang et al., 2019). Research has also indicated that CDT can induce immunogenic cell death, shifting the tumor microenvironment from a “cold” immunosuppression to a “hot” immunoactivation state (Xu et al., 2022). Furthermore, studies have shown that Cu^+^, acting as a catalyst in the Fenton reaction (Liu et al., 2022), has significant potential in combining cuproptosis induction strategies with CDT therapy. Li’s group developed a Cu-coordinated covalent organic framework Cu-COF integrating two valence copper ions. Cu^+^ can promote the generation of ·OH and ^1^O_2_ within cells, leading to DNA damage and lipid peroxidation. Additionally, Cu^2+^ can deplete endogenous GSH, further reducing the elimination of ROS and enhancing the Fenton-like effect produced by Cu^+^ (Li et al., 2023b). The combined effects of these functions can promote immunogenic cell death, and exert a potent anti-tumor effect. Xia et al. also developed a novel drug delivery strategy (LDH/HA/5-FU) to implement the CDT therapy based on Cu. Overexpressing GSH in TME can partially convert Cu^2+^ in LDH/HA/5-FU nanosheets to Cu^+^, the reduced Cu^+^ can act as an efficacious Fenton-like reaction catalyst and disrupt copper homeostasis within tumor cells, instigating the onset of cuproptosis. Furthermore, LDH/HA/5-FU nanosheets not only exhibit excellent biosafety but also can reconfigure TME as an immunomodulator to escalate the infiltration level of antitumor M1-like TAMs, CD4^+^ T cells and CD8^+^ T cells (Xia et al., 2024). By integrating CDT, chemotherapy, and immunotherapy, LDH/HA/5-FU nanosheets have displayed impressive potential in treating solid tumors. Recent research led by Fang et al. developed an injectable zwitterionic hydrogel system (Gel@M/CuO_2_/DOX/STING) loaded with nanoparticles (M/CuO_2_/DOX) and STING agonists 2′,3′-cGAMP, demonstrating a superior ability to specifically target and eradicate tumor cells while reshaping TMEs. On the one side, hydrophilic STING agonists activate the STING pathway, upregulate interferon signaling-related genes and reshape suppressive TMEs, thereby promoting dendritic cell maturation and augmenting tumor-specific CD8^+^T cell infiltration. On the other side, M/CuO_2_/DOX can target and eliminate tumor cells via DOX-induced DNA damage, immunogenic cell death, and cuproptosis (Fang et al., 2023). Collectively these studies foreshadow a promising future for photodynamic therapy and cuproptosis inducers in augmenting the efficacy of colorectal cancer immunotherapy.

### 6.5 Natural product-based cuproptosis-inducing nanomedicines

Contemporary breakthroughs with Chinese herb extracts in cancer treatment demonstrate great promise. Numerous clinical agents, including paclitaxel and camptothecin, are derived from natural products and draw focus due to their exclusive mechanisms, minimal toxicity, and multi-aim effects. They have exhibited successful expansion of applications and enhancement of cancer immunotherapies. Specifically, some natural products have demonstrated cuproptosis-inducing abilities. For instance, Eupalinolide B has been observed to inhibit pancreatic cancer cell proliferation, migration, and invasion via ROS generation and cuproptosis (Huang et al., 2024). Hence, leveraging the inherent benefits of Chinese herb extracts and integrating them with cuproptosis inducers to formulate more potent nanomedicines for synergistic promotion of cuproptosis could aid in developing novel strategies for cuproptosis induction. For instance, Lu et al. developed a self-amplified cuprated nanoparticle (Cel-Cu NPs) utilizing celastrol (Cel), a natural product isolated from medicinal plants. As a multifunctional copper ionophore, Cel aids in copper delivery to tumor cells, and decrease GSH level in cancer cells, bolstering the efficacy of cuproptosis. Cel-Cu NPs can further activate immunogenic cell death, promote dendritic cell maturation, and increase CTLs infiltration (Lu S. et al., 2024). In combination with anti-PD-L1 treatment, Cel-Cu NPs can reverse the immunosuppressive tumor microenvironment and efficiently eliminate metastatic tumors. Moreover, a hydrogel composed of glycyrrhizin (GA), Cu^2+^, and celastrol not only stimulates ROS production and accelerates apoptosis but also modulates the tumor microenvironment, promotes tumor-associated macrophages polarize to M1-TAM, and activates DC-mediated antigen presentation to stimulate T cell proliferation through chemodynamic therapy, thereby synergizing with anti-PD-L1 antibodies and demonstrating promising clinical translational potential (Wu et al., 2025). The robust tolerance of tumor cells towards oxidative stress decreases the efficacy of ES. Augmenting intracellular Cu concentration and disrupting redox homeostasis could be a valuable strategy to enhance the anti-tumor mechanism of ES. To this end, Wu et al. developed a ROS-responsive polymer (PCP) using cinnamaldehyde (CA), the primary component of cinnamon, to encapsulate ES-Cu compounds (EC) and form ECPCP. The PCP coating disintegrates in response to high ROS levels, releasing ES and Cu and inducing tumor cell cuproptosis. CA disrupts mitochondrial function and produces substantial ROS, triggering oxidative stress. Simultaneously, Cu^2+^-activated Fenton reaction and CA-induced ROS generation could disrupt the redox homeostasis and supplement the oxidative stress induced by ES, leading to ICD of tumor cells. The combined synergy of cuproptosis and immunotherapy presents a potential optimization approach for the clinical application of ES (Wu H. et al., 2024).

### 6.6 Multiple cell death pathway-targeted nanodrugs

The process of cancer cell death is a complex and carefully controlled process. Different modes of cell death are interconnected and can work together synergistically (Wang et al., 2024). These various modes of cell death may share common pathways and have a profound influence on each other. For example, the ferroptosis stimulators sorafenib and erastin have been shown to enhance copper-induced lipoacylated protein aggregation, leading to cuproptosis in primary liver cancer cells (Wang W. et al., 2023). Additionally, the combined administration of copper ionophores ES and Cu has been found to induce copper-dependent ferroptosis in colorectal cancer cells by breaking down ATP7A, resulting in copper accumulation within mitochondria (Gao et al., 2021). Understanding the interaction between different modes of cell death and their combination could lead to improved anti-tumor treatment outcomes. Qiao et al. designed self-destructive and multienzymatically active copper-quinone-GOx nanoparticles (CQG NPs), which can stimulate NLRP3-mediated pyroptosis while releasing self-destructive copper ions that induce cuproptosis. The liberated quinone further depletes the endogenous copper chelator GSH, increasing the vulnerability of cancer cells to cuproptosis. The simultaneous occurrence of these two modes of cell death can trigger the release of DAMPs, shift tumor-associated macrophages from an immunoinhibitory M2 phenotype to an immunostimulatory M1 phenotype, reverse the immunosuppressive TME, ignite a robust systemic immune response, greatly enhancing immune therapeutic effects, providing a novel strategy against tumor dormancy and preventing tumor relapse (Qiao et al., 2023). Li et al. synthesized a nanoreactor Cu_2-x_Se that binds the ferroptosis agonist erastin to the surface of Cu_2-x_Se in order to enhance intracellular oxygen utilization and GSH consumption, leading to ferroptosis through Cu^+^ accumulation. Furthermore, Cu_2-x_Se has the ability to modulate the phenotypic polarization of TAMs, increase IFN-γ production by CD8+T cells, and develop a combined therapeutic protocol that simultaneously activates ferroptosis, copper-dependent cell death, and anti-tumor immune responses (Li K. et al., 2023). Similarly, in another study, a core-shell nanoparticle CuP/Er was designed to incorporate copper and erastin into cancer cells to synergistically promote cuproptosis and ferroptosis. CuP/Er not only increase tumor cell sensitivity to cuproptosis, but also induces strong immunogenic cell death, enhances antigen presentation, and upregulates PD-L1 expression, thereby synergizing with anti-PD-L1 antibodies to effectively mediate regression of colon adenocarcinoma in mice (Li Y. et al., 2024).In conclusion, the advancement of therapeutic strategies targeting multiple cell death pathways will help overcome cancer cell resistance. Drugs targeting crucial proteins common to multiple cell death pathways may demonstrate superior efficacy compared with drugs focusing on a single cell death pathway.

### 6.7 Synergistic strategy of cuproptosis-inducing nanomedicines combined with radiotherapy and immunotherapy

Radiation therapy (RT) employs high-energy ionizing radiation to directly or indirectly damage DNA duplexes, inducing DNA double-strand breaks, and triggering diverse tumor cell death (Schaue and McBride, 2015). Radioimmunotherapy, driven by radiation therapy-induced ICD, is a promising approach to supplement the effect of conventional RT, which is limited to local tumor treatment. However, RT’s effective ICD activation is limited by radiation dose, weak tumor immunogenicity, and radioresistance due to the TME (Jarosz-Biej et al., 2019). The integration of cuproptosis-inducing nanomedicine with RT could potentially address these challenges. Cu^2+^ can deplete endogenous GSH and catalyze intratumoral H_2_O_2_ conversion to O_2_, thereby regulating tumor cell radioresistance. Besides, reduced Cu^+^ can generate highly toxic hydroxyl radicals (·OH) from H_2_O_2_, augmenting tumor oxidative stress, amplifying ICD, and shifting M2 TAMs to anti-tumor M1 TAMs. Additionally, tumor cell cuproptosis can stimulate ICD activation and enhance tumor immunogenicity. Hence, the RT-cuproptosis-immunotherapy cascade strategy holds significant promise in colorectal cancer. For instance, Jiang et al. designed a Cu-Hf dual-ion hybrid nanostimulator (CHP) for TME manipulation, achieving tumor radiosensitization at low X-ray doses, ROS accumulation, and cuproptosis promotion. Moreover, cuproptosis can bolster RT-induced anti-tumor immune responses via ICD activation, leading to robust anti-tumor immunity (Jiang et al., 2024). Similarly, Shen et al. engineered a sodium alginate hydrogel incorporating ES-Cu and galactose, which not only efficiently induces cuproptosis in colorectal tumor cells but also significantly enhancing the sensitivity of colorectal tumors to radiotherapy and immunotherapy (Shen W. et al., 2023). Furthermore, Li et al. developed a novel multifunctional copper-based nanocomposite (RCL@Pd @CuZ) to enhance RT sensitivity. This platform, with DSPE-PEG-RGD modification for tumor targeting and penetration, has been demonstrated to amplify ICD by improving hypoxia, promoting cuproptosis, depleting GSH, amplifying oxidative stress, and enhancing X-ray absorption. In mouse tumor models, RCL@Pd @CuZ + RT achieved >90% tumor growth inhibition compared with RT alone, and RCL@Pd @CuZ + RT + anti-PD-1 treatment maximally suppressed tumor growth (Li R. et al., 2024). These approach holds immense potential for leveraging cuproptosis to enhance tumor radioimmunotherapy.

## 7 Discussion

Copper plays a dual role in cellular processes. Maintaining optimal copper levels is crucial for cell survival and function, while excessive copper can induce cell death. Understanding the mechanism of cuproptosis opens up new possibilities for managing colorectal cancer. In colorectal cancer cells, the concentration of copper is significantly higher than that of normal cells, which means that they are more sensitive to cuproptosis. By delivering more copper into colorectal tumor cells, once the concentration of copper exceeds the scavenging ability of tumor cells, it triggers cuproptosis. The dual role of copper in cancer depends on its concentration and distribution within tumor cells. By precisely controlling the concentration of copper, it can be transformed into an effective anti-cancer strategy. Integrating cuproptosis induction strategies with existing treatment approaches may offer a breakthrough in overcoming tumor drug resistance. Recent bioinformatics studies have highlighted that key genes involved in cuproptosis could potentially enhance anti-tumor immunotherapy by influencing immune cell extravasation and immune checkpoint molecule expression within the TME. Moreover, cuproptosis may also improve the effectiveness of colorectal cancer immunotherapy by promoting immunogenic death. These findings underscore the significant potential of combining cuproptosis-inducing strategies with immunotherapy. However, further investigation is necessary before implementing this approach into clinical practice.

Directed delivery of copper ions to tumor cells is crucial for the development of effective cuproptosis-inducing therapies. However, direct delivery of copper ions is highly challenging due to their inherent instability. Furthermore, existing cuproptosis-inducing drugs have limited therapeutic efficacy due to drawbacks such as rapid excretion, poor bioavailability, and limited tumor accumulation. Additionally, they may also cause severe systemic toxicity and side effects when used directly. The invention of more efficient copper ion nano-drug delivery systems will help address these issues comprehensively. Nevertheless, while optimizing the physicochemical properties of nanomedicines is crucial for enhancing targeted therapeutic effects, it should be noted that incorporating nanoparticles may lead to unforeseen side effects. Thus, comprehensive histological and pharmacological analysis is necessary. Moreover, the inefficient accumulation and permeation of nano-drug delivery systems within tumor sites hinder the efficacy of cuproptosis-inducing nano-drugs significantly. Studies have shown that only 0.7% of drugs reach solid tumors after systemic administration (Challenging paradigms in tumour drug, 2020). Consequently, strategies to enhance the penetration and rapid diffusion of more nano-drugs across tumor blood vessels are imperative. Presently, most nano-drug delivery systems remain in the *in vitro* experimental phase, with poor in vitro-in vivo correlation. The therapeutic efficacy and safety in humans require further optimization. It is vital to extend the blood circulation time of nano-drugs and ensure their long-term biosafety. Hence, a detailed evaluation of nanomaterial’s pharmacokinetics, biodegradability, and metabolic pathways is necessary. Prospectively exploring the biological mechanism of cuproptosis in-depth and scientifically designing copper-based nanomedicines will provide a remarkably promising diagnostic and treatment strategy for colorectal malignancies.

Currently, research on cuproptosis is still in its early stages, with a predominant focus on bioinformatics studies. There remains a lack of comprehensive understanding regarding the molecular mechanism underlying cuproptosis. In future investigations, it is imperative to thoroughly explore the regulatory roles and pathways associated with cuproptosis in tumors while examining its involvement in tumor initiation, growth, metastasis, particularly in cancer stem cells and TME. Furthermore, it is essential to investigate potential mechanisms that hinder drug resistance against copper-based nanomedicines by exploring tumor cell self-regulation mechanisms related to intracellular copper homeostasis.

ROS plays a vital role in the manifestation of various forms of cell death. Strategically designed combination therapies aimed at activating multiple pathways of cell death associated with ROS will contribute to enhancing the efficacy of cancer treatment. For instance, therapeutic modalities such as radiotherapy, photodynamic therapy, chemokinetic therapy, and sonodynamic therapy have demonstrated their ability to induce significant levels of ROS, showing great potential for synergistic cuproptosis induction protocols and tumor eradication.

Another interesting phenomenon is lactate’s role in copper metabolism within the tumor microenvironment. Firstly, lactate accumulation results in TME acidification, thereby affecting copper ion bioavailability (Inaba and Takenaka, 2005). Secondly, heightened lactate levels can influence the functioning of copper transporters like CTR1 and ATP7A/B, thereby altering copper uptake and efflux in cells. Shen et al. discovered that heightened lactate levels could increase CTR1 expression mediated by Grhl2 via lactate modification of histone H3K18 in the Grhl2 promoter region, thereby promoting intracellular copper accumulation and enhancing cuproptosis (Shen et al., 2024). However, Zafar et al. reported lactate reduction markedly increased tumor cell susceptibility to cuproptosis (Zafar et al., 2024). Moreover, Menkes disease patient serum lactate levels (with ATP7A genetic deficiency) were substantially elevated, implying a potential connection between lactate and human copper metabolism (Gu et al., 2014). Concurrently, lactate may also regulate the redox potential by reducing the PH in the tumor microenvironment (Jamieson et al., 2015), influencing copper oxidation status, stimulating ROS production, and subsequently affecting copper metabolism via the oxidative stress pathway. In summary, the impact and mechanism of lactate in the tumor microenvironment on copper metabolism and cuproptosis require further investigation.

As previously mentioned, intracellular copper plays a crucial role in regulating the infiltration of diverse immune cells and the expression of PD-L1 in tumor cells, suggesting that copper dyshomeostasis can influence the immune system in a variety of ways. Tumor immunotherapy represents an innovative approach for cancer treatment, and advancements in nanoscience, including targeted delivery technology optimization, improved tumor vaccine design, and meticulous integration with PDT and CDT therapies are expected to further enhance colorectal cancer immunotherapy by inducing cuproptosis. Investigations into the functional significance of cuproptosis in tumor metabolism and immune response will establish a solid theoretical foundation for future development of more potent cuproptosis-inducing nanomedicine combined immunotherapy strategies.

To optimize the potential of cuproptosis-inducing nanomedicines in colorectal cancer immunotherapy, certain aspects in their formulation need to be clarified. The selection of copper ionophores is critical when preparing these medicines. Current popular choices include DSF and ES. ES is considered the more promising due to its superior delivery efficiency and mitochondria-specific Cu transport. ES can stimulate the production of ROS in cancer cells, induce mitochondrial oxidative stress, and endorse anti-tumor activity through cuproptosis induction (Gao et al., 2023). However, its limited tumor accumulation and rapid clearance in circulation limit its clinical applications. On the other hand, DSF possesses anti-cancer efficacy across various cancer types, and displays favorable safety in human trials (Kang et al., 2023). Both ionophores have relative merits, with their effectiveness depend on cancer type, stage, and patient traits. Hence, superiority cannot be unequivocally assigned. Future developmental endeavors can adapt the ionophore selection based on the unique cancer type and tumor metabolic features. Additionally, natural copper ionophores like curcumin and celastrol from Chinese herbs hold promise in the development of advanced cuproptosis-inducing nanomedicines. Besides, the size, structure and shape of cuproptosis-inducing NPs can substantially influence the circulation time, biodistribution, cellular uptake, and targetability of copper-based nanomaterials (Tang et al., 2014). The selection of nanomaterials in copper-based drug requires cautious deliberation. Nanomaterial include polymers, liposomes, and Cu-MOF have proven successful application in cancer treatment. Thus, it is wise to fully comprehend the intrinsic characteristics of various nanomaterials and integrate them with copper-based drugs to create an efficient colorectal cancer cuproptosis-inducing and immunotherapy synergistic nanoplatform. Besides, the choice of ICIs is equally significant. Presently, most cuproptosis-induction nanomedicines combined with ICIs select PD-L1 inhibitors. On one hand, copper accumulation can promote PD-L1 upregulation on tumor cells (Zhou et al., 2019), increase the binding checkpoints for PD-L1 inhibitors and enhancing their efficacy. On the other hand, PD-L1 is primarily expressed on tumor cells and antigen-presenting cells. PD-L1 inhibitors can directly block the interaction between PD-L1 on tumor cells and PD-1 on T cells, retaining the regulatory effect of PD-L2 on immune response, making them more specific in inhibiting tumor cell immune evasion. Further investigation is required on the actual effect of the combination of PD-1 inhibitors and cuproptosis-inducing nanomedicines. We also expect the exploration of synergistic therapy between other immune checkpoint molecules such as CTLA-4, LAG-3 inhibitors, and cuproptosis-inducing nanomedicines.

In the future, our research should focus on the following points. Firstly, we should concentrate on refining copper ionophores to enhance their tumor cell selectivity and copper ion transport efficiency. Secondly, ascertaining the optimum copper ion concentration for triggering cuproptosis in colorectal cancer cells necessitates the development of more accurate measures to measure copper levels in plasma and cells. This would help discerning and analyzing the therapeutic process of cuproptosis and facilitate the translation of cuproptosis-induced nanomedicines into clinical practice. Thirdly, devising novel nanomaterial to augment the targeting and specific accumulation of cuproptosis-inducing nanomedicines for colorectal cancer is necessary. Strategies such as modifying NPs’ surfaces with antibodies, peptides, proteins, and small molecules (e.g., folic acid, hyaluronic acid), can specifically bind to overexpressed receptors or antigens on cancer cells. This facilitates the internalization of NPs by cancer cells via receptor-mediated endocytosis, and allows for direct delivery of loaded active ingredients to tumor cells. Additionally, the utilization of biomimetic cell membranes for NP surface modification can enhance ligand binding and increase tumor cell uptake efficiency, achieving homologous tumor targeting (Fang et al., 2014).Furthermore, platelet membrane-coated NPs can be targeted by p-selectin on cell membranes to bind CD44 overexpressed in cancer cells, thus improving cancer cell targeting (Hu et al., 2015). Employing TME stimulation-responsive nano-delivery vehicles can acheive safe encapsulation of drugs and controlled drug release, facilitating cuproptosis-induced drug delivery to specific tumor sites and minimizing healthy tissue toxicity. Examples include pH-sensitive, ROS-activated, and GSH-responsive drugs, etc (Lu J. et al., 2024). It has been found that the TME of colorectal cancer is abundant in H_2_S, thus advocating the utilization of H_2_S-responsive copper nanoparticles for targeted colorectal cancer treatment (Lu J. et al., 2024). Fourthly, further study into the co-regulatory proteins and signaling pathways between cuproptosis and other cell death pathway is essential, and the development of multi-pathway targeted therapy can decrease drug resistance. Fifthly, oral cuproptosis-inducing nanomedicines for colorectal cancer treatment must to be developed. Lastly, the application of artificial intelligence technology coupled with the development of machine learning tools holds significant potential for optimizing cuproptosis-induced nanomedicine design.

## 8 Conclusion and future perspective

Colorectal cancer is a highly heterogeneous disease, posing significant challenges for clinicians due to its treatment resistance and metastatic potential. The emerging field of cuproptosis-based immunotherapy has garnered increasing attention as it offers promising approaches for overcoming drug resistance in CRC, controlling cancer metastasis, and enhancing the efficacy of immunotherapy. Particularly, nanodrugs capable of inducing cuproptosis have emerged as a transformative approach with their unique advantages including targeted delivery, multi-drug combination therapy, and synergistic multimodal strategies. Despite significant advancements in cuproptosis-inducing nanomedicines for anti-tumor immunotherapy, several issues necessitate careful consideration. Firstly, the immune response against tumors initiated by cuproptosis is partially mediated through immunogenic cell death. However, monotherapy-induced ICD often fails to elicit a sufficient immune response. Nanotechnology can synergistically enhance various treatment approaches, reversing the immunosuppressive tumor microenvironment and significantly augmenting the impact of anti-tumor immunotherapy. Secondly, further investigation is required to elucidate the mechanism and biological safety of cuproptosis-inducing nanomedicines combined with immunotherapy. Additionally, precise molecular typing is crucial for identifying a subgroup of CRC patients likely to positively respond to the combination of cuproptosis-inducing nanomedicine and immunotherapy, particularly those with dMMR/MSI-H phenotype. In addition to MMR and MSI status, additional reliable biomarkers are needed to predict the efficacy of this synergistic therapy.

Future research and development priorities for cuproptosis-based nanomedicines include enhancing their targeting capabilities, optimizing pharmacodynamic properties, increasing copper transport capacity and developing synergistic therapeutic strategies to enhance efficacy. The most crucial aspect is the precise delivery of copper ions through nanomedicine to minimize damage to normal tissues while utilizing cuproptosis as a cancer treatment. (He et al., 2024). Cuproptosis originates from mitochondrial copper overload; therefore, targeted mitochondrial nanodelivery systems have the potential to induce cuproptosis more effectively. Although an increased concentration of Cu^+^ promotes the elimination of tumor cells, the *in vivo* metabolism of nanomedicines and activation of copper excretion pathways gradually reduce the copper content within tumor cells. Simultaneously, slightly elevated levels of copper may facilitate tumor growth and metastasis. Therefore, it is crucial to engineer suitable nanomedicines that can modify their pharmacokinetic profiles in order to maintain a lethal level of copper ions within tumors. Lastly, the suboptimal outcome of colorectal cancer immunotherapy primarily stems from an immunosuppressive TME. Future efforts should focus on developing effective cuproptosis-inducing nanomedicines combined with immunotherapy strategies aimed at reversing this phenomenon.

In conclusion, the integration of cuproptosis-inducing nano-drug delivery system with immunotherapy represents a promising avenue for advancing research and clinical translation in colorectal cancer treatment, poised to emerge as a significant field of investigation. Given the rapid progress in nanotechnology, we anticipate that synergistic immunotherapy inducing cuproptosis will optimize the anti-tumor efficacy and ultimately serve as an optimal therapy for CRC.

## Glossary

CRC: Colorectal cancer
PD-1: Programmed cell death protein 1
PD-L1: Programmed death-ligand 1
ICIs: Immune checkpoint inhibitors
dMMR: Mismatch repair deficiency
MSI-H: High microsatellite instability
TME: Tumor microenvironment
DMT1: Dimethyltryptamine N-methyltransferase 1
CP: Ceruloplasmin
SOD: Superoxide dismutase
ROS: Reactive oxygen species
IMS: Intermembrane space
CuL: Copper ligands
SLC25A3: Solute carrier family 25 member 3
IMM: Inner mitochondrial membrane
CCO: Cytochrome-c oxidase,
CCS: Copper chaperone for superoxide dismutase 1
COMMD1: Copper metabolism Murr1 domain
XIAP: X-linked inhibitor of apoptosis proteins
RTK: Receptor Tyrosine Kinase
ERK: Extracellular regulated protein kinases
CSC: Cancer stem cell
IL-17: Interleukin-17
NF-κB: Nuclear factor kappa B
TGF-β: Transforming growth factor-β
ECM: Extracellular matrix
HIF-1: Hypoxia-inducible factor 1
FDX1: Ferredoxin 1
DLAT: Dihydrolipoamide S-acetyltransferase
TCA: Tricarboxylic acid
STEAP: Six-transmembrane epithelial antigen of the prostate
DCYTB: Duodenal cytochrome b
CTR1: Cu transport protein 1
SLC31A1: Solute carrier family 31 member 1
ATP7A: ATPase copper transporting alpha
MT1/2: Metallothionein1/2
GSH: Glutathione
SOD1: Superoxide dismutase 1
ATOX1: Antioxidant 1
TGN: Trans-Golgi network
COX: Cytochrome oxidase
MEK: Mitogen-activated extracellular signal-regulated kinase
MAPK: Mitogen-activated protein kinase
FGF: Fibroblast growth factors
VEGF: Vascular endothelial growth factor
TNF-α: Tumor necrosis factor alpha
ICD: Immunogenic cell death
NK cells: Natural killer cells
MDSCs: Myeloid-derived suppressor cells
TAMs: Tumor-associated macrophages
Tregs: Regulatory cells
ccRCC: Clear cell renal cell carcinoma
lncRNAs: Long non-coding RNAs
EMT: Epithelial-mesenchymal transition
CRLs: Cuproptosis-related lncRNAs
CRT: Calreticulin
DAMPs: Damage-associated molecular patterns
HMGB1: High-mobility group box 1 protein
PRRs: Pattern recognition receptors
OSCC: Oral squamous cell carcinoma
DC: Dendritic cell
CAR-T: chimeric antigen receptor T cells
PNPs: Polymer nanoparticles
PM: Polymer micelles
MOF: Metal-organic framework
NPs: Nanoparticles
TNBC: Triple negative breast cancer
CTL: Cytotoxic T lymphocyte
NIR: Near-infrared
CA: Cinnamaldehyde
RT: Radiation therapy
LIAS: Lipoic acid synthase
CDKN2A: Cyclin-dependent kinase inhibitor 2A
DSF: Disulfiram
ES: Elesclomol
PTT: Photothermal therapy
PDT: Photodynamic therapy
CDT: Chemodynamic therapy
SDT: Sonodynamic therapy
MELK: Maternal embryonic leucine zipper kinase
cGAS: Cyclic GMP-AMP synthase
STING: Stimulator of interferon genes

## References

[B1] Allensworth J. L. Evans M. K. Bertucci F. Aldrich A. J. Festa R. A. Finetti P. (2015). Disulfiram (DSF) acts as a copper ionophore to induce copper-dependent oxidative stress and mediate anti-tumor efficacy in inflammatory breast cancer. Mol. Oncol. 9 (6), 1155–1168. 10.1016/j.molonc.2015.02.007 25769405 PMC4493866

[B2] Babu U. Failla M. L. (1990). Copper status and function of neutrophils are reversibly depressed in marginally and severely copper-deficient rats. J. Nutr. 120 (12), 1700–1709. 10.1093/jn/120.12.1700 2175782

[B3] Bala S. Failla M. L. Lunney J. K. (1991). Alterations in splenic lymphoid cell subsets and activation antigens in copper-deficient rats. J. Nutr. 121 (5), 745–753. 10.1093/jn/121.5.745 1673467

[B4] Bandmann O. Weiss K. H. Kaler S. G. (2015). Wilson's disease and other neurological copper disorders. Lancet Neurology 14 (1), 103–113. 10.1016/S1474-4422(14)70190-5 25496901 PMC4336199

[B5] Baszuk P. Marciniak W. Derkacz R. Jakubowska A. Cybulski C. Gronwald J. (2021). Blood copper levels and the occurrence of colorectal cancer in Poland. Biomedicines 9 (11), 1628. 10.3390/biomedicines9111628 34829856 PMC8615693

[B6] Beach M. A. Nayanathara U. Gao Y. Zhang C. Xiong Y. Wang Y. (2024). Polymeric nanoparticles for drug delivery. Chem. Rev. 124 (9), 5505–5616. 10.1021/acs.chemrev.3c00705 38626459 PMC11086401

[B7] Bian C. Zheng Z. Su J. Chang S. Yu H. Bao J. (2023). Copper homeostasis and cuproptosis in tumor pathogenesis and therapeutic strategies. Front. Pharmacol. 14, 1271613. 10.3389/fphar.2023.1271613 37767404 PMC10520736

[B8] Boyd S. D. Ullrich M. S. Skopp A. Winkler D. D. (2020). Copper sources for Sod1 activation. Antioxidants Basel, Switz. 9 (6), 500. 10.3390/antiox9060500 PMC7346115 32517371

[B9] Brady D. C. Crowe M. S. Greenberg D. N. (2017). Counter CM copper chelation inhibits BRAF(V600e)-driven melanomagenesis and counters resistance to BRAF(V600E) and MEK1/2 inhibitors. Cancer Res. 77 (22), 6240–6252. 10.1158/0008-5472.CAN-16-1190 28986383 PMC5690876

[B10] Cao Y. Langer R. Ferrara N. (2023). Targeting angiogenesis in oncology, ophthalmology and beyond. Nat. Rev. Drug Discov. 22 (6), 476–495. 10.1038/s41573-023-00671-z 37041221

[B11] Cater M. A. La Fontaine S. Shield K. Deal Y. Mercer J. F. (2006). ATP7B mediates vesicular sequestration of copper insight into biliary copper excretion. Gastroenterology 130 (2), 493–506. 10.1053/j.gastro.2005.10.054 16472602

[B12] Cervantes A. Adam R. Roselló S. Arnold D. Normanno N. Taïeb J. (2023). Metastatic colorectal cancer ESMO Clinical Practice Guideline for diagnosis, treatment and follow-up. Ann. Oncol. official J. Eur. Soc. Med. Oncol. 34 (1), 10–32. 10.1016/j.annonc.2022.10.003 36307056

[B13] Challenging paradigms in tumour drug delivery. Nat. Mater. 2020, 19(5)477. 10.1038/s41563-020-0676-x 32332992

[B14] Chan W. Y. Garnica A. D. Rennert O. M. (1978). Cell culture studies of Menkes kinky hair disease. Clin. chimica acta; Int. J. Clin. Chem. 88 (3), 495–507. 10.1016/0009-8981(78)90284-x 699339

[B15] Chen J. Jiang Y. Shi H. Peng Y. Fan X. Li C. (2020). The molecular mechanisms of copper metabolism and its roles in human diseases. Pflugers Archiv Eur. J. physiology 472 (10), 1415–1429. 10.1007/s00424-020-02412-2 32506322

[B16] Chen K. Zhou A. Zhou X. Liu Y. Xu Y. Ning X. (2023c). An intelligent cell-derived nanorobot bridges synergistic crosstalk between sonodynamic therapy and cuproptosis to promote cancer treatment. Nano Lett. 23 (7), 3038–3047. 10.1021/acs.nanolett.3c00434 36951267

[B17] Chen L. Min J. Wang F. (2022). Copper homeostasis and cuproptosis in health and disease. Signal Transduct. Target. Ther. 7 (1), 378. 10.1038/s41392-022-01229-y 36414625 PMC9681860

[B18] Chen S. Shen X. (2020). Long noncoding RNAs functions and mechanisms in colon cancer. Mol. cancer 19 (1), 167. 10.1186/s12943-020-01287-2 33246471 PMC7697375

[B19] Chen X. Cai Q. Liang R. Zhang D. Liu X. Zhang M. (2023b). Copper homeostasis and copper-induced cell death in the pathogenesis of cardiovascular disease and therapeutic strategies. Cell. death and Dis. 14 (2), 105. 10.1038/s41419-023-05639-w PMC9922317 36774340

[B20] Chen Z. Li Y. Y. Liu X. (2023a). Copper homeostasis and copper-induced cell death novel targeting for intervention in the pathogenesis of vascular aging. Biomed. and Pharmacother. = Biomedecine and Pharmacother. 169, 115839. 10.1016/j.biopha.2023.115839 37976889

[B21] Cheng F. Peng G. Lu Y. Wang K. Ju Q. Ju Y. (2022). Relationship between copper and immunity the potential role of copper in tumor immunity. Front. Oncol. 12, 1019153. 10.3389/fonc.2022.1019153 36419894 PMC9676660

[B22] Chojnacka M. Gornicka A. Oeljeklaus S. Warscheid B. Chacinska A. (2015). Cox17 protein is an auxiliary factor involved in the control of the mitochondrial contact site and cristae organizing system. J. Biol. Chem. 290 (24), 15304–15312. 10.1074/jbc.M115.645069 25918166 PMC4463469

[B23] Chowrimootoo G. F. Seymour C. A. (1994). The role of caeruloplasmin in copper excretion. Biochem. Soc. Trans. 22 (2), 190s. 10.1042/bst022190s 7958253

[B24] Chu B. Wang Y. Yang J. Dong B. (2023). Integrative analysis of single-cell and bulk RNA seq to reveal the prognostic model and tumor microenvironment remodeling mechanisms of cuproptosis-related genes in colorectal cancer. Aging 15 (23), 14422–14444. 10.18632/aging.205324 38078879 PMC10756095

[B25] Coan M. Haefliger S. Ounzain S. Johnson R. (2024). Targeting and engineering long non-coding RNAs for cancer therapy. Nat. Rev. Genet. 25, 578–595. 10.1038/s41576-024-00693-2 38424237

[B26] Copyright © 1993-2024 (1993). GeneReviews is a registered trademark of the University of Washington, Seattle. University of Washington, Seattle. All rights reserved.

[B27] Cox D. W. Moore S. D. (2002). Copper transporting P-type ATPases and human disease. J. bioenergetics Biomembr. 34 (5), 333–338. 10.1023/a1021293818125 12539960

[B28] Demaria O. Cornen S. Daëron M. Morel Y. Medzhitov R. Vivier E. (2019). Harnessing innate immunity in cancer therapy. Nature 574 (7776), 45–56. 10.1038/s41586-019-1593-5 31578484

[B29] Deng R. H. Zou M. Z. Zheng D. Peng S. Y. Liu W. Bai X. F. (2019). Nanoparticles from cuttlefish ink inhibit tumor growth by synergizing immunotherapy and photothermal therapy. ACS nano 13 (8), 8618–8629. 10.1021/acsnano.9b02993 31246413

[B30] Deng W. Shang H. Tong Y. Liu X. Huang Q. He Y. (2024). The application of nanoparticles-based ferroptosis, pyroptosis and autophagy in cancer immunotherapy. J. nanobiotechnology 22 (1), 97. 10.1186/s12951-024-02297-8 38454419 PMC10921615

[B31] De Silva M. Tse B. C. Y. Diakos C. I. Clarke S. Molloy M. P. (2024). Immunogenic cell death in colorectal cancer a review of mechanisms and clinical utility. Cancer Immunol. Immunother. CII 73 (3), 53. 10.1007/s00262-024-03641-5 38353760 PMC10866783

[B32] Díez-Tercero L. Delgado L. M. Perez R. A. (2022). Modulation of macrophage response by copper and magnesium ions in combination with low concentrations of dexamethasone. Biomedicines 10 (4), 764. 10.3390/biomedicines10040764 35453514 PMC9030383

[B33] Ding K. Mou P. Wang Z. Liu S. Liu J. Lu H. (2023). The next bastion to be conquered in immunotherapy microsatellite stable colorectal cancer. Front. Immunol. 14, 1298524. 10.3389/fimmu.2023.1298524 38187388 PMC10770832

[B34] Dreishpoon M. B. Bick N. R. Petrova B. Warui D. M. Cameron A. Booker S. J. (2023). FDX1 regulates cellular protein lipoylation through direct binding to LIAS. J. Biol. Chem. 299 (9), 105046. 10.1016/j.jbc.2023.105046 37453661 PMC10462841

[B35] Erez N. (2015). Cancer opening LOX to metastasis. Nature 522 (7554), 41–42. 10.1038/nature14529 26017311

[B36] Fadahunsi A. A. Li C. Khan M. I. Ding W. (2022). MXenes state-of-the-art synthesis, composites and bioapplications. J. Mater. Chem. B 10 (23), 4331–4345. 10.1039/d2tb00289b 35640492

[B37] Fang R. H. Hu C. M. Luk B. T. Gao W. Copp J. A. Tai Y. (2014). Cancer cell membrane-coated nanoparticles for anticancer vaccination and drug delivery. Nano Lett. 14 (4), 2181–2188. 10.1021/nl500618u 24673373 PMC3985711

[B38] Fang Y. Huang S. Hu Q. Zhang J. King J. A. Wang Y. (2023). Injectable zwitterionic physical hydrogel with enhanced chemodynamic therapy and tumor microenvironment remodeling properties for synergistic anticancer therapy. ACS nano 17 (24), 24883–24900. 10.1021/acsnano.3c05898 37883579

[B39] Farasatkia A. Maeso L. Gharibi H. Dolatshahi-Pirouz A. Stojanovic G. M. Edmundo Antezana P. (2024). Design of nanosystems for melanoma treatment. Int. J. Pharm. 665, 124701. 10.1016/j.ijpharm.2024.124701 39278291

[B40] Franke A. J. Skelton W. P. Starr J. S. Parekh H. Lee J. J. Overman M. J. (2019). Immunotherapy for colorectal cancer a review of current and novel therapeutic approaches. J. Natl. Cancer Inst. 111 (11), 1131–1141. 10.1093/jnci/djz093 31322663 PMC6855933

[B41] Gajewski T. F. Schreiber H. Fu Y. X. (2013). Innate and adaptive immune cells in the tumor microenvironment. Nat. Immunol. 14 (10), 1014–1022. 10.1038/ni.2703 24048123 PMC4118725

[B42] Galamb O. Barták B. K. Kalmár A. Nagy Z. B. Szigeti K. A. Tulassay Z. (2019). Diagnostic and prognostic potential of tissue and circulating long non-coding RNAs in colorectal tumors. World J. gastroenterology 25 (34), 5026–5048. 10.3748/wjg.v25.i34.5026 PMC6747286 31558855

[B43] Ganetzky R. McCormick E. M. Falk M. J., 2024, Primary pyruvate dehydrogenase complex deficiency overview In Edited by Adam M. P. Feldman J. Mirzaa G. M. Pagon R. A. Wallace S. E. Bean L. J. H. Seattle (WA) University of Washington, Seattle 34138529

[B44] Gao J. Wu X. Huang S. Zhao Z. He W. Song M. (2023). Novel insights into anticancer mechanisms of elesclomol more than a prooxidant drug. Redox Biol. 67, 102891. 10.1016/j.redox.2023.102891 37734229 PMC10518591

[B45] Gao L. Zhang A. (2023). Copper-instigated modulatory cell mortality mechanisms and progress in oncological treatment investigations. Front. Immunol. 14, 1236063. 10.3389/fimmu.2023.1236063 37600774 PMC10433393

[B46] Gao W. Huang Z. Duan J. Nice E. C. Lin J. Huang C. (2021). Elesclomol induces copper-dependent ferroptosis in colorectal cancer cells via degradation of ATP7A. Mol. Oncol. 15 (12), 3527–3544. 10.1002/1878-0261.13079 34390123 PMC8637554

[B47] Ge E. J. Bush A. I. Casini A. Cobine P. A. Cross J. R. DeNicola G. M. (2022). Connecting copper and cancer from transition metal signalling to metalloplasia. Nat. Rev. Cancer 22 (2), 102–113. 10.1038/s41568-021-00417-2 34764459 PMC8810673

[B48] Grochowski C. Blicharska E. Baj J. Mierzwińska A. Brzozowska K. Forma A. (2019). Serum iron, magnesium, copper, and manganese levels in alcoholism a systematic review. Mol. Basel, Switz. 24 (7), 1361. 10.3390/molecules24071361 PMC6480471 30959950

[B49] Gu Y. H. Kodama H. Ogawa E. Izumi Y. (2014). Lactate and pyruvate levels in blood and cerebrospinal fluid in patients with Menkes disease. J. Pediatr. 164 (4), 890–894. 10.1016/j.jpeds.2013.11.045 24388330

[B50] Guan D. Zhao L. Shi X. Ma X. Chen Z. (2023). Copper in cancer from pathogenesis to therapy. Biomed. and Pharmacother. 163, 114791. 10.1016/j.biopha.2023.114791 37105071

[B51] Guo B. Yang F. Zhang L. Zhao Q. Wang W. Yin L. (2023b). Cuproptosis induced by ROS responsive nanoparticles with elesclomol and copper combined with αpd-L1 for enhanced cancer immunotherapy. Adv. Mater. Deerf. Beach, Fla 35 (22), e2212267. 10.1002/adma.202212267 36916030

[B52] Guo L. Wang C. Qiu X. Pu X. Chang P. (2020). Colorectal cancer immune infiltrates significance in patient prognosis and immunotherapeutic efficacy. Front. Immunol. 11, 1052. 10.3389/fimmu.2020.01052 32547556 PMC7270196

[B53] Guo P. Niu Z. Zhang D. Zhao F. Li J. Lu T. (2023a). Potential impact of cuproptosis-related genes on tumor immunity in esophageal carcinoma. Aging 15 (24), 15535–15556. 10.18632/aging.205391 38159255 PMC10781504

[B54] Gupta A. Lutsenko S. (2009). Human copper transporters mechanism, role in human diseases and therapeutic potential. Future Med. Chem. 1 (6), 1125–1142. 10.4155/fmc.09.84 20454597 PMC2863355

[B55] Halama N. Braun M. Kahlert C. Spille A. Quack C. Rahbari N. (2011). Natural killer cells are scarce in colorectal carcinoma tissue despite high levels of chemokines and cytokines. Clin. cancer Res. official J. Am. Assoc. Cancer Res. 17 (4), 678–689. 10.1158/1078-0432.CCR-10-2173 21325295

[B56] Hari S. K. Gauba A. Shrivastava N. Tripathi R. M. Jain S. K. Pandey A. K. (2023). Polymeric micelles and cancer therapy an ingenious multimodal tumor-targeted drug delivery system. Drug Deliv. Transl. Res. 13 (1), 135–163. 10.1007/s13346-022-01197-4 35727533

[B57] Hasan N. M. Gupta A. Polishchuk E. Yu C. H. Polishchuk R. Dmitriev O. Y. (2012). Molecular events initiating exit of a copper-transporting ATPase ATP7B from the trans-Golgi network. J. Biol. Chem. 287 (43), 36041–36050. 10.1074/jbc.M112.370403 22898812 PMC3476272

[B58] He F. Chang C. Liu B. Li Z. Li H. Cai N. (2019). Copper (II) ions activate ligand-independent receptor tyrosine kinase (RTK) signaling pathway. BioMed Res. Int. 2019, 4158415. 10.1155/2019/4158415 31218225 PMC6537018

[B59] He T. Tang Q. Ren Q. Liu Y. He G. Pan Y. (2024). Different valence states of copper ion delivery against triple-negative breast cancer. ACS nano. 10.1021/acsnano.3c10226 38320291

[B60] Helmink B. A. Reddy S. M. Gao J. Zhang S. Basar R. Thakur R. (2020). B cells and tertiary lymphoid structures promote immunotherapy response. Nature 577 (7791), 549–555. 10.1038/s41586-019-1922-8 31942075 PMC8762581

[B61] Hu H. Xu Q. Mo Z. Hu X. He Q. Zhang Z. (2022). New anti-cancer explorations based on metal ions. J. nanobiotechnology 20 (1), 457. 10.1186/s12951-022-01661-w 36274142 PMC9590139

[B62] Hu Q. Sun W. Qian C. Wang C. Bomba H. N. Gu Z. (2015). Anticancer platelet-mimicking nanovehicles. Adv. Mater. Deerf. Beach, Fla 27 (44), 7043–7050. 10.1002/adma.201503323 PMC4998740 26416431

[B63] Hu X. Zhao W. Li R. Chai K. Shang F. Shi S. (2023). A cascade nanoplatform for the regulation of the tumor microenvironment and combined cancer therapy. Nanoscale 15 (40), 16314–16322. 10.1039/d3nr03199c 37786260

[B64] Hu Y. Qian Y. Wei J. Jin T. Kong X. Cao H. (2021). The disulfiram/copper complex induces autophagic cell death in colorectal cancer by targeting ULK1. Front. Pharmacol. 12, 752825. 10.3389/fphar.2021.752825 34887757 PMC8650091

[B65] Huang Q. Yang J. Zhang J. Yao L. Jiang B. Du S. (2024). Eupalinolide B suppresses pancreatic cancer by ROS generation and potential cuproptosis. iScience 27 (8), 110496. 10.1016/j.isci.2024.110496 39100694 PMC11295471

[B66] Huang Q.-X. Liang J.-L. Chen Q.-W. Jin X.-K. Niu M.-T. Dong C.-Y. (2023). Metal-organic framework nanoagent induces cuproptosis for effective immunotherapy of malignant glioblastoma. Nano Today 51, 101911. 10.1016/j.nantod.2023.101911

[B67] Inaba S. Takenaka C. (2005). Effects of dissolved organic matter on toxicity and bioavailability of copper for lettuce sprouts. Environ. Int. 31 (4), 603–608. 10.1016/j.envint.2004.10.017 15788200

[B68] Itoh S. Kim H. W. Nakagawa O. Ozumi K. Lessner S. M. Aoki H. (2008). Novel role of antioxidant-1 (Atox1) as a copper-dependent transcription factor involved in cell proliferation. J. Biol. Chem. 283 (14), 9157–9167. 10.1074/jbc.M709463200 18245776 PMC2431038

[B69] Jamieson L. E. Jaworska A. Jiang J. Baranska M. Harrison D. J. Campbell C. J. (2015). Simultaneous intracellular redox potential and pH measurements in live cells using SERS nanosensors. Analyst 140 (7), 2330–2335. 10.1039/c4an02365j 25700000

[B70] Jarosz-Biej M. Smolarczyk R. Cichoń T. Kułach N. (2019). Tumor microenvironment as A game changer in cancer radiotherapy. Int. J. Mol. Sci. 20 (13), 3212. 10.3390/ijms20133212 31261963 PMC6650939

[B71] Jiang A. Luo P. Chen M. Fang Y. Liu B. Wu Z. (2022). A new thinking deciphering the aberrance and clinical implication of copper-death signatures in clear cell renal cell carcinoma. Cell. and Biosci. 12 (1), 209. 10.1186/s13578-022-00948-7 PMC9801655 36581992

[B72] Jiang X. Wang J. Huang W. Ma H. Zhang S. Cai Z. (2024). Tumor microenvironment reprogrammed bimetallic hybrid nanostimulator for triggering radio-cuproptosis-immunotherapy. Adv. Healthc. Mater., e2401902. 10.1002/adhm.202401902 39136059

[B73] Jiang Z. Sha G. Zhang W. Zhang Z. Liu T. Wang D. (2023). The huge potential of targeting copper status in the treatment of colorectal cancer. Clin. and Transl. Oncol. official Publ. Fed. Span. Oncol. Soc. Natl. Cancer Inst. Mexico 25 (7), 1977–1990. 10.1007/s12094-023-03107-7 36781599

[B74] Kaler S. G. (2011). ATP7A-related copper transport diseases-emerging concepts and future trends. Nat. Rev. Neurol. 7 (1), 15–29. 10.1038/nrneurol.2010.180 21221114 PMC4214867

[B75] Kang D. E. Lim C. S. Kim J. Y. Kim E. S. Chun H. J. Cho B. R. (2014). Two-photon probe for Cu^2^⁺ with an internal reference quantitative estimation of Cu^2^⁺ in human tissues by two-photon microscopy. Anal. Chem. 86 (11), 5353–5359. 10.1021/ac500329k 24825103

[B76] Kang X. Jadhav S. Annaji M. Huang C. H. Amin R. Shen J. (2023). Advancing cancer therapy with copper/disulfiram nanomedicines and drug delivery systems. Pharmaceutics 15 (6), 1567. 10.3390/pharmaceutics15061567 37376016 PMC10302862

[B77] Kardos J. Héja L. Simon Á. Jablonkai I. Kovács R. Jemnitz K. (2018). Copper signalling causes and consequences. Cell. Commun. Signal. CCS 16 (1), 71. 10.1186/s12964-018-0277-3 30348177 PMC6198518

[B78] Kaur P. Johnson A. Northcote-Smith J. Lu C. Suntharalingam K. (2020). Immunogenic cell death of breast cancer stem cells induced by an endoplasmic reticulum-targeting copper(II) complex. Chembiochem a Eur. J. Chem. Biol. 21 (24), 3618–3624. 10.1002/cbic.202000553 PMC7757018 32776422

[B79] Kim J. E. Jeon S. Lindahl P. A. (2023). Discovery of an unusual copper homeostatic mechanism in yeast cells respiring on minimal medium and an unexpectedly diverse labile copper pool. J. Biol. Chem. 299 (12), 105435. 10.1016/j.jbc.2023.105435 37944620 PMC10704325

[B80] Krężel A. Maret W. (2017). The functions of metamorphic metallothioneins in zinc and copper metabolism. Int. J. Mol. Sci. 18 (6), 1237. 10.3390/ijms18061237 28598392 PMC5486060

[B81] Ladomersky E. Petris M. J. (2015). Copper tolerance and virulence in bacteria. Metallomics Integr. biometal Sci. 7 (6), 957–964. 10.1039/c4mt00327f PMC4464932 25652326

[B82] Li H. van der Merwe P. A. Sivakumar S. (2022a). Biomarkers of response to PD-1 pathway blockade. Br. J. cancer 126 (12), 1663–1675. 10.1038/s41416-022-01743-4 35228677 PMC9174485

[B83] Li K. Xu K. He Y. Yang Y. Tan M. Mao Y. (2023c). Oxygen self-generating nanoreactor mediated ferroptosis activation and immunotherapy in triple-negative breast cancer. ACS nano 17 (5), 4667–4687. 10.1021/acsnano.2c10893 36861638

[B84] Li Q. Chao Y. Liu B. Xiao Z. Yang Z. Wu Y. (2022b). Disulfiram loaded calcium phosphate nanoparticles for enhanced cancer immunotherapy. Biomaterials 291, 121880. 10.1016/j.biomaterials.2022.121880 36334355

[B85] Li R. Zhao W. Han Z. Feng N. Wu T. Xiong H. (2024b). Self-cascade nanozyme reactor as a cuproptosis inducer synergistic inhibition of cellular respiration boosting radioimmunotherapy. Small Weinheim der Bergstrasse, Ger. 20 (25), e2306263. 10.1002/smll.202306263 38221757

[B86] Li T. Wang D. Meng M. Yang Z. Luo Z. Li Z. (2023b). Copper-coordinated covalent organic framework produced a robust fenton-like effect inducing immunogenic cell death of tumors. Macromol. rapid Commun. 44 (11), e2200929. 10.1002/marc.202200929 36840703

[B87] Li T. Zhang Y. Zhu J. Zhang F. Xu A. Zhou T. (2023a). A pH-activatable copper-biomineralized proenzyme for synergistic chemodynamic/chemo-immunotherapy against aggressive cancers. Adv. Mater. Deerf. Beach, Fla 35 (14), e2210201. 10.1002/adma.202210201 36573375

[B88] Li X. Lovell J. F. Yoon J. Chen X. (2020). Clinical development and potential of photothermal and photodynamic therapies for cancer. Nat. Rev. Clin. Oncol. 17 (11), 657–674. 10.1038/s41571-020-0410-2 32699309

[B89] Li Y. Liu J. Chen Y. Weichselbaum R. R. Lin W. (2024a). Nanoparticles synergize ferroptosis and cuproptosis to potentiate cancer immunotherapy. Adv. Sci. Weinheim, Baden-Wurttemberg, Ger. 11 (23), e2310309. 10.1002/advs.202310309 PMC11187894 38477411

[B90] Liang W. Han C. Zhang D. Liu C. Zhu M. Xu F. (2024). Copper-coordinated nanoassemblies based on photosensitizer-chemo prodrugs and checkpoint inhibitors for enhanced apoptosis-cuproptosis and immunotherapy. Acta biomater. 175, 341–352. 10.1016/j.actbio.2023.12.022 38122883

[B91] Liao Y. Zhao J. Bulek K. Tang F. Chen X. Cai G. (2020). Inflammation mobilizes copper metabolism to promote colon tumorigenesis via an IL-17-STEAP4-XIAP axis. Nat. Commun. 11 (1), 900. 10.1038/s41467-020-14698-y 32060280 PMC7021685

[B92] Lin X. Kang K. Chen P. Zeng Z. Li G. Xiong W. (2024). Regulatory mechanisms of PD-1/PD-L1 in cancers. Mol. cancer 23 (1), 108. 10.1186/s12943-024-02023-w 38762484 PMC11102195

[B93] Linder M. C. (2001). Copper and genomic stability in mammals. Mutat. Res. 475 (1-2), 141–152. 10.1016/s0027-5107(01)00076-8 11295159

[B94] Linder M. C. Hazegh-Azam M. (1996). Copper biochemistry and molecular biology. Am. J. Clin. Nutr. 63 (5), 797s–811s. 10.1093/ajcn/63.5.797 8615367

[B95] Linder M. C. Wooten L. Cerveza P. Cotton S. Shulze R. Lomeli N. (1998). Copper transport. Am. J. Clin. Nutr. 67 (5 Suppl. l), 965s–971s. 10.1093/ajcn/67.5.965S 9587137

[B96] Liochev S. I. Fridovich I. (2002). The Haber-Weiss cycle -- 70 years later an alternative view. Redox Rep. Commun. free Radic. Res. 7 (1), 55–60. 10.1179/135100002125000190 11981456

[B97] Liu F. Ou W. Tang W. Huang Z. Zhu Z. Ding W. (2021). Increased AOC1 expression promotes cancer progression in colorectal cancer. Front. Oncol. 11, 657210. 10.3389/fonc.2021.657210 34026633 PMC8131869

[B98] Liu P. Peng Y. Ding J. Zhou W. (2022). Fenton metal nanomedicines for imaging-guided combinatorial chemodynamic therapy against cancer. Asian J. Pharm. Sci. 17 (2), 177–192. 10.1016/j.ajps.2021.10.003 35582641 PMC9091802

[B99] Liu T. Zhou Z. Zhang M. Lang P. Li J. Liu Z. (2023b). Cuproptosis-immunotherapy using PD-1 overexpressing T cell membrane-coated nanosheets efficiently treats tumor. J. Control. release official J. Control. Release Soc. 362, 502–512. 10.1016/j.jconrel.2023.08.055 37652367

[B100] Liu Y. Hu Y. Xue J. Li J. Yi J. Bu J. (2023a). Advances in immunotherapy for triple-negative breast cancer. Mol. cancer 22 (1), 145. 10.1186/s12943-023-01850-7 37660039 PMC10474743

[B101] Liu Y. Niu R. Zhang X. Zhang B. Chen X. Guo J. (2024b). Metal-organic framework-based nanovaccine for relieving immunosuppressive tumors via hindering efferocytosis of macrophages and promoting pyroptosis and cuproptosis of cancer cells. ACS nano 18 (19), 12386–12400. 10.1021/acsnano.4c01518 38699808

[B102] Liu Y. T. Chen L. Li S. J. Wang W. Y. Wang Y. Y. Yang Q. C. (2024a). Dysregulated Wnt/β-catenin signaling confers resistance to cuproptosis in cancer cells. Cell. death Differ. 31, 1452–1466. 10.1038/s41418-024-01341-2 38987382 PMC11520902

[B103] Lopez J. Ramchandani D. Vahdat L. (2019). Copper depletion as a therapeutic strategy in cancer. Metal ions life Sci. 19. 10.1515/9783110527872-018 30855113

[B104] Lu J. Miao Y. Li Y. (2024c). Cuproptosis advances in stimulus-responsive nanomaterials for cancer therapy. Adv. Healthc. Mater. 13 (19), e2400652. 10.1002/adhm.202400652 38622782

[B105] Lu S. Li Y. Yu Y. (2024b). Glutathione-scavenging celastrol-Cu nanoparticles induce self-amplified cuproptosis for augmented cancer immunotherapy. Adv. Mater. Deerf. Beach, Fla 36 (35), e2404971. 10.1002/adma.202404971 38935977

[B106] Lu X. Chen X. Lin C. Yi Y. Zhao S. Zhu B. (2024a). Elesclomol loaded copper oxide nanoplatform triggers cuproptosis to enhance antitumor immunotherapy. Adv. Sci. Weinheim, Baden-Wurttemberg, Ger. 11 (18), e2309984. 10.1002/advs.202309984 PMC11095170 38430531

[B107] Lu Y. Pan Q. Gao W. Pu Y. Luo K. He B. (2022). Leveraging disulfiram to treat cancer mechanisms of action, delivery strategies, and treatment regimens. Biomaterials 281, 121335. 10.1016/j.biomaterials.2021.121335 34979419

[B108] Lutsenko S. (2021). Dynamic and cell-specific transport networks for intracellular copper ions. J. Cell. Sci. 134 (21), jcs240523. 10.1242/jcs.240523 34734631 PMC8627558

[B109] Lutsenko S. Barnes N. L. Bartee M. Y. Dmitriev O. Y. (2007). Function and regulation of human copper-transporting ATPases. Physiol. Rev. 87 (3), 1011–1046. 10.1152/physrev.00004.2006 17615395

[B110] Lutsenko S. Roy S. Tsvetkov P. (2024). Mammalian copper homeostasis physiologic roles and molecular mechanisms. Physiol. Rev. 10.1152/physrev.00011.2024 PMC11918410 39172219

[B111] Mo J. Q. Zhang S. Y. Li Q. Chen M. X. Zheng Y. Q. Xie X. (2024). Immunomodulation of cuproptosis and ferroptosis in liver cancer. Cancer Cell. Int. 24 (1), 22. 10.1186/s12935-023-03207-y 38200525 PMC10777659

[B112] Munk D. E. Vendelbo M. H. Kirk F. T. Rewitz K. S. Bender D. A. Vase K. H. (2023). Distribution of non-ceruloplasmin-bound copper after i.v. (64)Cu injection studied with PET/CT in patients with Wilson disease. JHEP Rep. innovation hepatology 5 (11), 100916. 10.1016/j.jhepr.2023.100916 PMC10597763 37886434

[B113] Myint Z. W. Oo T. H. Thein K. Z. Tun A. M. Saeed H. (2018). Copper deficiency anemia review article. Ann. Hematol. 97 (9), 1527–1534. 10.1007/s00277-018-3407-5 29959467

[B114] Neeser A. Ramasubramanian R. Wang C. Ma L. (2023). Engineering enhanced chimeric antigen receptor-T cell therapy for solid tumors. Immuno-oncology Technol. 19, 100385. 10.1016/j.iotech.2023.100385 PMC10362352 37483659

[B115] Nývltová E. Dietz J. V. Seravalli J. Khalimonchuk O. Barrientos A. (2022). Coordination of metal center biogenesis in human cytochrome c oxidase. Nat. Commun. 13 (1), 3615. 10.1038/s41467-022-31413-1 35750769 PMC9232578

[B116] O'Day S. J. Eggermont A. M. Chiarion-Sileni V. Kefford R. Grob J. J. Mortier L. (2013). Final results of phase III SYMMETRY study randomized, double-blind trial of elesclomol plus paclitaxel versus paclitaxel alone as treatment for chemotherapy-naive patients with advanced melanoma. J. Clin. Oncol. official J. Am. Soc. Clin. Oncol. 31 (9), 1211–1218. 10.1200/JCO.2012.44.5585 23401447

[B117] Oliveri V. (2022). Selective targeting of cancer cells by copper ionophores an overview. Front. Mol. Biosci. 9, 841814. 10.3389/fmolb.2022.841814 35309510 PMC8931543

[B118] Ortega M. A. Boaru D. L. De Leon-Oliva D. Fraile-Martinez O. García-Montero C. Rios L. (2024). PD-1/PD-L1 axis implications in immune regulation, cancer progression, and translational applications. J. Mol. Med. 102 (8), 987–1000. 10.1007/s00109-024-02463-3 38935130

[B119] Oshi M. Asaoka M. Tokumaru Y. Angarita F. A. Yan L. Matsuyama R. (2020). Abundance of regulatory T cell (treg) as a predictive biomarker for neoadjuvant chemotherapy in triple-negative breast cancer. Cancers 12 (10), 3038. 10.3390/cancers12103038 33086518 PMC7603157

[B120] Palumaa P. (2013). Copper chaperones. The concept of conformational control in the metabolism of copper. FEBS Lett. 587 (13), 1902–1910. 10.1016/j.febslet.2013.05.019 23684646

[B121] Pan H. Liu P. Zhao L. Pan Y. Mao M. Kroemer G. (2024). Immunogenic cell stress and death in the treatment of cancer. Seminars Cell. and Dev. Biol. 156, 11–21. 10.1016/j.semcdb.2023.10.007 37977108

[B122] Pan Q. Xie L. Liu R. Pu Y. Wu D. Gao W. (2022). Two birds with one stone copper metal-organic framework as a carrier of disulfiram prodrug for cancer therapy. Int. J. Pharm. 612, 121351. 10.1016/j.ijpharm.2021.121351 34883206

[B123] Pang L. Wang Q. Wang L. Hu Z. Yang C. Li Y. (2023). Development and validation of cuproptosis-related lncRNA signatures for prognosis prediction in colorectal cancer. BMC Med. genomics 16 (1), 58. 10.1186/s12920-023-01487-x 36949429 PMC10031908

[B124] Prakash A. Gates T. Zhao X. Wangmo D. Subramanian S. (2023). Tumor-derived extracellular vesicles in the colorectal cancer immune environment and immunotherapy. Pharmacol. and Ther. 241, 108332. 10.1016/j.pharmthera.2022.108332 36526013

[B125] Prohaska J. R. (2008). Role of copper transporters in copper homeostasis. Am. J. Clin. Nutr. 88 (3), 826s–9S. 10.1093/ajcn/88.3.826S 18779302 PMC2799992

[B126] Prohaska J. R. Lukasewycz O. A. (1981). Copper deficiency suppresses the immune response of mice. Sci. (New York, NY) 213 (4507), 559–561. 10.1126/science.7244654 7244654

[B127] Qiao L. Zhu G. Jiang T. Qian Y. Sun Q. Zhao G. (2023). Self-destructive copper carriers induce pyroptosis and cuproptosis for efficient tumor immunotherapy against dormant and recurrent tumors. Adv. Mater. Deerf. Beach, Fla 36, e2308241. 10.1002/adma.202308241 37820717

[B128] Qin Y. Liu Y. Xiang X. Long X. Chen Z. Huang X. (2023). Cuproptosis correlates with immunosuppressive tumor microenvironment based on pan-cancer multiomics and single-cell sequencing analysis. Mol. cancer 22 (1), 59. 10.1186/s12943-023-01752-8 36959665 PMC10037895

[B129] Rae T. D. Schmidt P. J. Pufahl R. A. Culotta V. C. O'Halloran T. V. (1999). Undetectable intracellular free copper the requirement of a copper chaperone for superoxide dismutase. Sci. (New York, NY) 284 (5415), 805–808. 10.1126/science.284.5415.805 10221913

[B130] Roelofsen H. Wolters H. Van Luyn M. J. Miura N. Kuipers F. Vonk R. J. (2000). Copper-induced apical trafficking of ATP7B in polarized hepatoma cells provides a mechanism for biliary copper excretion. Gastroenterology 119 (3), 782–793. 10.1053/gast.2000.17834 10982773

[B131] Ruan Y. Zhuang H. Zeng X. Lin L. Wang X. Xue P. (2024). Engineered microbial nanohybrids for tumor-mediated NIR II photothermal enhanced ferroptosis/cuproptosis and immunotherapy. Adv. Healthc. Mater. 13 (4), e2302537. 10.1002/adhm.202302537 37742322

[B132] Russell J. H. Ley T. J. (2002). Lymphocyte-mediated cytotoxicity. Annu. Rev. Immunol. 20, 323–370. 10.1146/annurev.immunol.20.100201.131730 11861606

[B133] Ruturaj M. M. Saha S. Maji S. Rodriguez-Boulan E. Schreiner R. Gupta A. (2024). Regulation of the apico-basolateral trafficking polarity of the homologous copper-ATPases ATP7A and ATP7B. J. Cell. Sci. 137 (5), jcs261258. 10.1242/jcs.261258 38032054 PMC10729821

[B134] Sadeghi M. Dehnavi S. Sharifat M. Amiri A. M. Khodadadi A. (2024). Innate immune cells key players of orchestra in modulating tumor microenvironment (TME). Heliyon 10 (5), e27480. 10.1016/j.heliyon.2024.e27480 38463798 PMC10923864

[B135] Sailer J. Nagel J. Akdogan B. Jauch A. T. Engler J. Knolle P. A. (2024). Deadly excess copper. Redox Biol. 75, 103256. 10.1016/j.redox.2024.103256 38959622 PMC11269798

[B136] Schaue D. McBride W. H. (2015). Opportunities and challenges of radiotherapy for treating cancer. Nat. Rev. Clin. Oncol. 12 (9), 527–540. 10.1038/nrclinonc.2015.120 26122185 PMC8396062

[B137] Shanbhag V. Jasmer-McDonald K. Zhu S. Martin A. L. Gudekar N. Khan A. (2019). ATP7A delivers copper to the lysyl oxidase family of enzymes and promotes tumorigenesis and metastasis. Proc. Natl. Acad. Sci. U. S. A. 116 (14), 6836–6841. 10.1073/pnas.1817473116 30890638 PMC6452744

[B138] Shanbhag V. C. Gudekar N. Jasmer K. Papageorgiou C. Singh K. Petris M. J. (2021). Copper metabolism as a unique vulnerability in cancer. Biochimica biophysica acta Mol. Cell. Res. 1868 (2), 118893. 10.1016/j.bbamcr.2020.118893 PMC7779655 33091507

[B139] Sharifi L. Nowroozi M. R. Amini E. Arami M. K. Ayati M. Mohsenzadegan M. (2019). A review on the role of M2 macrophages in bladder cancer; pathophysiology and targeting. Int. Immunopharmacol. 76, 105880. 10.1016/j.intimp.2019.105880 31522016

[B140] Shawki A. Anthony S. R. Nose Y. Engevik M. A. Niespodzany E. J. Barrientos T. (2015). Intestinal DMT1 is critical for iron absorption in the mouse but is not required for the absorption of copper or manganese. Am. J. physiology Gastrointest. liver physiology 309 (8), G635–G647. 10.1152/ajpgi.00160.2015 PMC4609933 26294671

[B141] Shen S. Gao Y. Ouyang Z. Jia B. Shen M. Shi X. (2023a). Photothermal-triggered dendrimer nanovaccines boost systemic antitumor immunity. J. Control. release official J. Control. Release Soc. 355, 171–183. 10.1016/j.jconrel.2023.01.076 36736909

[B142] Shen W. Pei P. Zhang C. Li J. Han X. Liu T. (2023b). A polymeric hydrogel to eliminate programmed death-ligand 1 for enhanced tumor radio-immunotherapy. ACS nano 17 (23), 23998–24011. 10.1021/acsnano.3c08875 37988029

[B143] Shen X. Y. Huang J. Chen L. L. Sha M. T. Gao J. Xin H. (2024). Blocking lactate regulation of the Grhl2/SLC31A1 axis inhibits trophoblast cuproptosis and preeclampsia development. J. assisted reproduction Genet. 10.1007/s10815-024-03256-w PMC11621273 39287710

[B144] Song Y. Tan K. B. Zhou S. F. Zhan G. (2024). Biocompatible copper-based nanocomposites for combined cancer therapy. ACS biomaterials Sci. and Eng. 10 (6), 3673–3692. 10.1021/acsbiomaterials.4c00586 38717176

[B145] Sun Z. Zhao H. Ma L. Shi Y. Ji M. Sun X. (2024). The quest for nanoparticle-powered vaccines in cancer immunotherapy. J. nanobiotechnology 22 (1), 61. 10.1186/s12951-024-02311-z 38355548 PMC10865557

[B146] Sung H. Ferlay J. Siegel R. L. Laversanne M. Soerjomataram I. Jemal A. (2021). Global cancer statistics 2020 GLOBOCAN estimates of incidence and mortality worldwide for 36 cancers in 185 countries. CA a cancer J. Clin. 71 (3), 209–249. 10.3322/caac.21660 33538338

[B147] Tang L. Yang X. Yin Q. Cai K. Wang H. Chaudhury I. (2014). Investigating the optimal size of anticancer nanomedicine. Proc. Natl. Acad. Sci. U. S. A. 111 (43), 15344–15349. 10.1073/pnas.1411499111 25316794 PMC4217425

[B148] Tang W. Wu J. Wang L. Wei K. Pei Z. Gong F. (2024). Bioactive layered double hydroxides for synergistic sonodynamic/cuproptosis anticancer therapy with elicitation of the immune response. ACS nano 18 (15), 10495–10508. 10.1021/acsnano.3c11818 38556991

[B149] Tang X. Yan Z. Miao Y. Ha W. Li Z. Yang L. (2023). Copper in cancer from limiting nutrient to therapeutic target. Front. Oncol. 13, 1209156. 10.3389/fonc.2023.1209156 37427098 PMC10327296

[B150] Tang Z. Liu Y. He M. Bu W. Therapy C. (2019). Chemodynamic therapy tumour microenvironment-mediated Fenton and fenton-like reactions. Angewandte Chemie Int. ed Engl. 58 (4), 946–956. 10.1002/anie.201805664 30048028

[B151] Tapiero H. Townsend D. M. Tew K. D. (2003). Trace elements in human physiology and pathology. Copper. Copp. Biomed. and Pharmacother. = Biomedecine and Pharmacother. 57 (9), 386–398. 10.1016/s0753-3322(03)00012-x PMC6361146 14652164

[B152] Tian Z. Jiang S. Zhou J. Zhang W. (2023). Copper homeostasis and cuproptosis in mitochondria. Life Sci. 334, 122223. 10.1016/j.lfs.2023.122223 38084674

[B153] Tsvetkov P. Coy S. Petrova B. Dreishpoon M. Verma A. Abdusamad M. (2022). Copper induces cell death by targeting lipoylated TCA cycle proteins. Sci. (New York, NY) 375 (6586), 1254–1261. 10.1126/science.abf0529 PMC9273333 35298263

[B154] Tury S. Chauveau L. Lecante A. Courgnaud V. Battini J. L. (2023). A co-opted endogenous retroviral envelope promotes cell survival by controlling CTR1-mediated copper transport and homeostasis. Cell. Rep. 42 (9), 113065. 10.1016/j.celrep.2023.113065 37682705

[B155] Umansky V. Blattner C. Gebhardt C. Utikal J. (2016). The role of myeloid-derived suppressor cells (MDSC) in cancer progression. Vaccines 4 (4), 36. 10.3390/vaccines4040036 27827871 PMC5192356

[B156] Vitaliti A. De Luca A. Rossi L. (2022). Copper-dependent kinases and their role in cancer inception, progression and metastasis. Biomolecules 12 (10), 1520. 10.3390/biom12101520 36291728 PMC9599708

[B157] Vo T. T. T. Peng T.-Y. Nguyen T. H. Bui T. N. H. Wang C.-S. Lee W.-J. (2024). The crosstalk between copper-induced oxidative stress and cuproptosis a novel potential anticancer paradigm. Cell. Commun. Signal. 22 (1), 353. 10.1186/s12964-024-01726-3 38970072 PMC11225285

[B158] Voli F. Valli E. Lerra L. Kimpton K. Saletta F. Giorgi F. M. (2020). Intratumoral copper modulates PD-L1 expression and influences tumor immune evasion. Cancer Res. 80 (19), 4129–4144. 10.1158/0008-5472.CAN-20-0471 32816860

[B159] Wang F. Lau J. K. C. Yu J. (2021). The role of natural killer cell in gastrointestinal cancer killer or helper. Oncogene 40 (4), 717–730. 10.1038/s41388-020-01561-z 33262461 PMC7843415

[B160] Wang J. Li J. Liu J. Chan K. Y. Lee H. S. Lin K. N. (2024). Interplay of ferroptosis and cuproptosis in cancer dissecting metal-driven mechanisms for therapeutic potentials. Cancers 16 (3), 512. 10.3390/cancers16030512 38339263 PMC10854932

[B161] Wang L. Cao Y. Guo W. Xu J. (2023d). High expression of cuproptosis-related gene FDX1 in relation to good prognosis and immune cells infiltration in colon adenocarcinoma (COAD). J. cancer Res. Clin. Oncol. 149 (1), 15–24. 10.1007/s00432-022-04382-7 36173462 PMC9889456

[B162] Wang W. Lu K. Jiang X. Wei Q. Zhu L. Wang X. (2023f). Ferroptosis inducers enhanced cuproptosis induced by copper ionophores in primary liver cancer. J. Exp. and Clin. cancer Res. CR 42 (1), 142. 10.1186/s13046-023-02720-2 37277863 PMC10242978

[B163] Wang X. Wu M. Li H. Jiang J. Zhou S. Chen W. (2022). Enhancing penetration ability of semiconducting polymer nanoparticles for sonodynamic therapy of large solid tumor. Adv. Sci. Weinheim, Baden-Wurttemberg, Ger. 9 (6), e2104125. 10.1002/advs.202104125 PMC8867194 34989170

[B164] Wang X. Zhang L. (2018). Kinetic study of hydroxyl radical formation in a continuous hydroxyl generation system. RSC Adv. 8 (71), 40632–40638. 10.1039/c8ra08511k 35557884 PMC9091360

[B165] Wang X. Zhou M. Liu Y. Si Z. (2023a). Cope with copper from copper linked mechanisms to copper-based clinical cancer therapies. Cancer Lett. 561, 216157. 10.1016/j.canlet.2023.216157 37011869

[B166] Wang Y. Drum D. L. Sun R. Zhang Y. Yu L. Jia L. (2023e). Stressed target cancer cells drive nongenetic reprogramming of CAR T cells and tumor microenvironment, overcoming multiple obstacles of CAR T therapy for solid tumors. Res. square. 10.21203/rs.3.rs-2595410/v1 PMC10504259 37714830

[B167] Wang Y. Zhong X. He X. Hu Z. Huang H. Chen J. (2023c). Liver metastasis from colorectal cancer pathogenetic development, immune landscape of the tumour microenvironment and therapeutic approaches. J. Exp. and Clin. cancer Res. CR 42 (1), 177. 10.1186/s13046-023-02729-7 37480104 PMC10362774

[B168] Wang Z. Jin D. Zhou S. Dong N. Ji Y. An P. (2023b). Regulatory roles of copper metabolism and cuproptosis in human cancers. Front. Oncol. 13, 1123420. 10.3389/fonc.2023.1123420 37035162 PMC10076572

[B169] Wen M. H. Xie X. Huang P. S. Yang K. Chen T. Y. (2021). Crossroads between membrane trafficking machinery and copper homeostasis in the nerve system. Open Biol. 11 (12), 210128. 10.1098/rsob.210128 34847776 PMC8633785

[B170] Wu H. Zhang Z. Cao Y. Hu Y. Li Y. Zhang L. (2024b). A self-amplifying ROS-responsive nanoplatform for simultaneous cuproptosis and cancer immunotherapy. Adv. Sci. Weinheim, Baden-Wurttemberg, Ger. 11 (23), e2401047. 10.1002/advs.202401047 PMC11187900 38569217

[B171] Wu L. Lin H. Cao X. Tong Q. Yang F. Miao Y. (2024a). Bioorthogonal Cu single-atom nanozyme for synergistic nanocatalytic therapy, photothermal therapy, cuproptosis and immunotherapy. Angewandte Chemie Int. ed Engl. 63 (27), e202405937. 10.1002/anie.202405937 38654446

[B172] Wu L. Pi W. Huang X. Yang L. Zhang X. Lu J. (2025). Orchestrated metal-coordinated carrier-free celastrol hydrogel intensifies T cell activation and regulates response to immune checkpoint blockade for synergistic chemo-immunotherapy. Biomaterials 312, 122723. 10.1016/j.biomaterials.2024.122723 39121732

[B173] Wu Z. Lv G. Xing F. Xiang W. Ma Y. Feng Q. (2023). Copper in hepatocellular carcinoma a double-edged sword with therapeutic potentials. Cancer Lett. 571, 216348. 10.1016/j.canlet.2023.216348 37567461

[B174] Xia Y. Gu M. Wang J. Zhang X. Shen T. Shi X. (2024). Tumor microenvironment-activated, immunomodulatory nanosheets loaded with copper(II) and 5-FU for synergistic chemodynamic therapy and chemotherapy. J. colloid interface Sci. 653 (Pt A), 137–147. 10.1016/j.jcis.2023.09.042 37713912

[B175] Xiong Y. Wang Y. Tiruthani K. (2019). Tumor immune microenvironment and nano-immunotherapeutics in colorectal cancer. Nanomedicine Nanotechnol. Biol. Med. 21, 102034. 10.1016/j.nano.2019.102034 31207314

[B176] Xu N. Hu A. Pu X. Wang J. Liao X. Huang Z. (2022). Cu-Chelated polydopamine nanoparticles as a photothermal medium and “immunogenic cell death” inducer for combined tumor therapy. J. Mater. Chem. B 10 (16), 3104–3118. 10.1039/d2tb00025c 35348176

[B177] Yang L. Zhang Y. Wang Y. Jiang P. Liu F. Feng N. (2022a). Ferredoxin 1 is a cuproptosis-key gene responsible for tumor immunity and drug sensitivity a pan-cancer analysis. Front. Pharmacol. 13, 938134. 10.3389/fphar.2022.938134 36210836 PMC9532935

[B178] Yang Y. Wang X. Lu J. Dong Z. Hu R. Chen W. (2023). Construction of a prognostic model for predicting colorectal cancer prognosis and response to immunotherapy based on cuproptosis-associated lncRNAs. J. Oncol. 2023, 2733232. 10.1155/2023/2733232 36968641 PMC10033210

[B179] Yang Z. Tao D. Zhong W. Liu Z. Feng L. Chen M. (2022b). Perfluorocarbon loaded fluorinated covalent organic polymers with effective sonosensitization and tumor hypoxia relief enable synergistic sonodynamic-immunotherapy. Biomaterials 280, 121250. 10.1016/j.biomaterials.2021.121250 34823883

[B180] Yatim N. Cullen S. Albert M. L. (2017). Dying cells actively regulate adaptive immune responses. Nat. Rev. Immunol. 17 (4), 262–275. 10.1038/nri.2017.9 28287107

[B181] Yu L. Liu Z. Xu W. Jin K. Liu J. Zhu X. (2024b). Towards overcoming obstacles of type II photodynamic therapy endogenous production of light, photosensitizer, and oxygen. Acta Pharm. Sin. B 14 (3), 1111–1131. 10.1016/j.apsb.2023.11.007 38486983 PMC10935104

[B182] Yu N. Zhou J. Ding M. Li M. Peng S. Li J. (2024a). Sono-triggered cascade lactate depletion by semiconducting polymer nanoreactors for cuproptosis-immunotherapy of pancreatic cancer. Angewandte Chemie Int. ed Engl. 63 (30), e202405639. 10.1002/anie.202405639 38708791

[B183] Zafar H. Zhang J. Raza F. Pan X. Hu Z. Feng H. (2024). Biomimetic gold nanocages incorporating copper-human serum albumin for tumor immunotherapy via cuproptosis-lactate regulation. J. Control. release official J. Control. Release Soc. 372, 446–466. 10.1016/j.jconrel.2024.06.059 38917953

[B184] Zeng J. Chen H. Liu X. Xia H. Chen L. Lin D. (2024). Cuproptosis in microsatellite stable colon cancer cells affects the cytotoxicity of CD8(+)T through the WNT signaling pathway. Chemico-biological Interact. 403, 111239. 10.1016/j.cbi.2024.111239 39306268

[B185] Zhang J. Peng L. Hao Y. Yang H. Zhao W. Mao C. (2023). Biodegradable CuMoO(4) nanodots with multienzyme activities for multimodal treatment of tumor. Adv. Healthc. Mater. 12 (22), e2300167. 10.1002/adhm.202300167 37223944

[B186] Zhao H. J. Zhao X. H. (2019). Modulatory effect of the supplemented copper ion on *in vitro* activity of bovine lactoferrin to murine splenocytes and RAW264.7 macrophages. Biol. trace Elem. Res. 189 (2), 519–528. 10.1007/s12011-018-1472-1 30117046

[B187] Zhao Y. Zhao B. Zhu S. (2024). Disulfiram/copper activates ER stress to promote immunogenic cell death of oral squamous cell carcinoma. Cell. Biochem. biophysics 82 (2), 1291–1298. 10.1007/s12013-024-01283-z 38727783

[B188] Zheng X. Liu Z. Mi M. Wen Q. Wu G. Zhang L. (2021). Disulfiram improves the anti-PD-1 therapy efficacy by regulating PD-L1 expression via epigenetically reactivation of IRF7 in triple negative breast cancer. Front. Oncol. 11, 734853. 10.3389/fonc.2021.734853 34858816 PMC8631359

[B189] Zheng Z. Zhang J. Jiang J. He Y. Zhang W. Mo X. (2020). Remodeling tumor immune microenvironment (TIME) for glioma therapy using multi-targeting liposomal codelivery. J. Immunother. cancer 8 (2), e000207. 10.1136/jitc-2019-000207 32817393 PMC7437977

[B190] Zhou B. Guo L. Zhang B. Liu S. Zhang K. Yan J. (2019). Disulfiram combined with copper induces immunosuppression via PD-L1 stabilization in hepatocellular carcinoma. Am. J. cancer Res. 9 (11), 2442–2455. 31815045 PMC6895448

[B191] Zhu Z. Zhao Q. Song W. Weng J. Li S. Guo T. (2022). A novel cuproptosis-related molecular pattern and its tumor microenvironment characterization in colorectal cancer. Front. Immunol. 13, 940774. 10.3389/fimmu.2022.940774 36248908 PMC9561547

[B192] Zununi Vahed S. Salehi R. Davaran S. Sharifi S. (2017). Liposome-based drug co-delivery systems in cancer cells. Mater. Sci. and Eng. C, Mater. Biol. Appl. 71, 1327–1341. 10.1016/j.msec.2016.11.073 27987688

